# Molecular Evolutionary Analyses of the RNA-Dependent RNA Polymerase (*RdRp*) Region and *VP1* Gene in Human Norovirus Genotypes GII.P6-GII.6 and GII.P7-GII.6

**DOI:** 10.3390/v15071497

**Published:** 2023-07-01

**Authors:** Tomoko Takahashi, Ryusuke Kimura, Tatsuya Shirai, Mitsuru Sada, Toshiyuki Sugai, Kosuke Murakami, Kazuhiko Harada, Kazuto Ito, Yuki Matsushima, Fuminori Mizukoshi, Kaori Okayama, Yuriko Hayashi, Mayumi Kondo, Tsutomu Kageyama, Yoshiyuki Suzuki, Haruyuki Ishii, Akihide Ryo, Kazuhiko Katayama, Kiyotaka Fujita, Hirokazu Kimura

**Affiliations:** 1 Department of Health Science, Graduate School of Health Sciences, Gunma Paz University, Takasaki-shi, Gunma 370-0006, Japan; tomo-takahashi@pref.iwate.jp (T.T.); okayama@paz.ac.jp (K.O.); hayashi@paz.ac.jp (Y.H.); fujita@paz.ac.jp (K.F.); 2 Iwate Prefectural Research Institute for Environmental Science and Public Health, Morioka-shi, Iwate 020-0857, Japan; 3 Advanced Medical Science Research Center, Gunma Paz University Research Institute, Shibukawa-shi, Gunma 377-0008, Japan; m2220015@gunma-u.ac.jp (R.K.); shirait28@gmail.com (T.S.); rainbow_orchestra716@yahoo.co.jp (M.S.); k_harada@bishinkai.or.jp (K.H.); kzito@gunma-u.ac.jp (K.I.); 4 Department of Bacteriology, Graduate School of Medicine, Gunma University, Maebashi-shi, Gunma 371-8514, Japan; 5 Department of Respiratory Medicine, School of Medicine, Kyorin University, Mitaka-shi, Tokyo 181-8611, Japan; h141@ks.kyorin-u.ac.jp; 6 Department of Nursing Science, Graduate School of Health Science, Hiroshima University, Hiroshima-shi, Hiroshima 734-8551, Japan; tsugai@hiroshima-u.ac.jp; 7 Department of Virology II, National Institute of Infectious Diseases, Musashimurayama-shi, Tokyo 208-0011, Japan; ko-mura@niid.go.jp; 8 Caliciviruses Section, Laboratory of Infectious Diseases, National Institute of Allergy and Infectious Diseases, National Institutes of Health, Bethesda, MD 20892, USA; yuki4649m780@gmail.com; 9 Department of Microbiology, Tochigi Prefectural Institute of Public Health and Environmental Science, Utsunomiya-shi, Tochigi 329-1196, Japan; mizukoshif01@pref.tochigi.lg.jp; 10 Department of Clinical Engineering, Faculty of Medical Technology, Gunma Paz University, Takasaki-shi, Gunma 370-0006, Japan; kondo@paz.ac.jp; 11 Center for Emergency Preparedness and Response, National Institute of Infectious Diseases, Musashimurayama-shi, Tokyo 208-0011, Japan; tkage@niid.go.jp; 12 Division of Biological Science, Department of Information and Basic Science, Graduate School of Natural Sciences, Nagoya City University, Nagoya-shi, Aichi 467-8501, Japan; yossuzuk@nsc.nagoya-cu.ac.jp; 13 Department of Virology III, National Institute of Infectious Diseases, Musashimurayama-shi, Tokyo 208-0011, Japan; aryo@niid.go.jp; 14 Laboratory of Viral Infection Control, Graduate School of Infection Control Sciences, Ōmura Satoshi Memorial Institute, Kitasato University, Minato-ku, Tokyo 108-8641, Japan; katayama@lisci.kitasato-u.ac.jp

**Keywords:** human norovirus, RNA-dependent RNA polymerase (*RdRp*) region, *VP1* gene, epitope mapping, molecular evolution

## Abstract

To understand the evolution of GII.P6-GII.6 and GII.P7-GII.6 strains, the prevalent human norovirus genotypes, we analysed both the *RdRp* region and *VP1* gene in globally collected strains using authentic bioinformatics technologies. A common ancestor of the P6- and P7-type *RdRp* region emerged approximately 50 years ago and a common ancestor of the P6- and P7-type *VP1* gene emerged approximately 110 years ago. Subsequently, the *RdRp* region and *VP1* gene evolved. Moreover, the evolutionary rates were significantly faster for the P6-type *RdRp* region and *VP1* gene than for the P7-type *RdRp* region and *VP1* genes. Large genetic divergence was observed in the P7-type *RdRp* region and *VP1* gene compared with the P6-type *RdRp* region and *VP1* gene. The phylodynamics of the *RdRp* region and *VP1* gene fluctuated after the year 2000. Positive selection sites in VP1 proteins were located in the antigenicity-related protruding 2 domain, and these sites overlapped with conformational epitopes. These results suggest that the GII.6 *VP1* gene and VP1 proteins evolved uniquely due to recombination between the P6- and P7-type *RdRp* regions in the HuNoV GII.P6-GII.6 and GII.P7-GII.6 virus strains.

## 1. Introduction

Human norovirus (HuNoV) is a major causative agent of acute gastroenteritis in humans of all ages [1,2]. Previous epidemiological data suggest that HuNoV may be associated with 30–60% of patients with gastroenteritis [3,4,5]. Moreover, this agent has caused large outbreaks of food poisoning worldwide [6,7]. However, effective vaccines and antiviral agents are not available at present [7]. Therefore, this agent may be a public health concern [8].

The HuNoV genome is a single-stranded plus-sense RNA with an approximately 7.5 kb nucleotide sequence [9]. The genome contains three open reading frames (ORFs): ORF1, ORF2, and ORF3 [9]. ORF1 encodes six nonstructural proteins designated as nonstructural proteins (NS) 1/2–7 [9]. Of these, the NS7 region encodes the RNA-dependent RNA polymerase (RdRp) protein, while ORF2 and ORF3 encode structural proteins, such as viral protein (VP) 1 and VP2, respectively [7,9]. The VP1 protein acts as an antigen and also shows large antigenic variations [6], although it is not exactly known.

Previous genetic and molecular epidemiological studies have suggested that the HuNoV genome shows large genetic divergence [8]. Currently, HuNoV is classified into three genogroups: genogroup I (GI), genogroup II (GII), and genogroup IV (GIV), with many genotypes based on the genetic divergence of *VP1* [10]. Due to the relatively frequent recombination between ORF1 and ORF2 [11,12], dual nomenclatures such as GII.P6 (*RdRp* genotype)-GII.6 (*VP1* genotype) have been used, utilizing both the *RdRp* region and *VP1* gene for genotyping [10,13]. To date, 60 and 49 types of *RdRp* (P-types) and *VP1* genotypes have been identified, respectively [10]. Moreover, genogroups and genotypes may be associated with disease severity [14]. Furthermore, recombination between ORF1 and ORF2 has resulted in many chimeric viruses [12]. However, the role of these chimeric viruses remains unknown. 

Molecular epidemiological data on HuNoV infections in humans suggest that certain GI and GII genotypes are prevalent [15]. These reports also show that GII HuNoV is more dominant than GI HuNoV [15]. Of these, some GII genotypes corresponding to *VP1* genotypes, such as GII.2, GII.3, GII.4, GII.6, and GII.17, are prevalent types [16,17,18,19]. However, these epidemiological data may not explain the reasons for the HuNoV epidemics. 

Recently, authentic bioinformatic technologies have been used in population genetics, including the study of the evolution of various viruses [20]. Indeed, these methods may allow us to estimate the phylogeny, genome population, and antigenicity using three-dimensional antigen structures. Information that reflects viral evolution may contribute to a better response to these questions. We studied the molecular evolution of chimeric HuNoV, such as GII.P17-GII.17, GII.P2-GII.2, and GII.P16-GII.2, which have caused major outbreaks in many countries [21,22,23]. However, such studies have not been performed on other GII genotypes to better understand GII HuNoV. In norovirus infections, GII.4 is the predominant genotype worldwide. However, recombinant GII.6 strains have been circulating since the 1970s, with outbreaks reported in Japan in 2008–2009 and in the United States and Italy in 2014–2015, with an overall prevalence second only to GII.4 [18,24,25]. Although this genotype has been reported to play an important epidemiological role in norovirus outbreaks, the molecular epidemiological mechanism underlying these outbreaks has not been studied in detail. Moreover, GII.6 almost always displays P6- or P7-type *RdRp* genotypes [26]. Therefore, in this study, we performed a comprehensive molecular analysis of globally collected HuNoV GII.P6-GII.6 and GII.P7-GII.6 strains.

## 2. Materials and Methods

### 2.1. Strains Used in This Study

To analyse the molecular evolution of HuNoV GII.6, the complete genome sequences of HuNoV were downloaded from GenBank (last accessed on 28 December 2022). In total, 11,810 strains were collected. They were classified into genotypes using the Norovirus Typing Tool (Ver.2.0), and GII.6 strains were selected [10]. HuNoV GII.6 data collected from each local government public health institution were added to the dataset because the number of GII.6 strains available for analysis, especially GII.P6-GII.6 strains, was small. Strains with an uncertain sequence or an unclear year of collection or area were excluded. Finally, 141 strains belonging to GII.6 remained and were used to analyse the molecular evolution of VP1. Similarly, 141 strains belonging to HuNoV GII.6 were obtained and used to analyse the molecular evolution of *RdRp* region. Details of the strains used in this study are presented in Supplementary Table S1.

### 2.2. Time-Scaled Phylogenetic Analyses

To evaluate the molecular evolution of the present strains, phylogenetic trees of the HuNoV *RdRp* region (1530 bp, excluding the stop codon) and the *VP1* gene (1641–1650 bp, excluding the stop codon) were constructed using the Bayesian Markov chain Monte Carlo (MCMC) method in the BEAST package (v.2.6.7), as previously described [27,28]. First, the jModelTest2 program was used to determine the suitable substitution models [29]. Second, the path-sampling/stepping-stone sampling marginal likelihood estimation method was used to evaluate the best of the four clock models (strict clock, relaxed clock exponential, relaxed clock log normal, and random local clock) and the two prior tree models (coalescent constant population and coalescent exponential population). Although these were performed independently for *VP1* gene and *RdRp* region analyses, SYM-Γ-I, relaxed clock exponential, and coalescent exponential population were selected for the molecular evolutionary analysis of *VP1* gene. However, SYM-Γ, relaxed clock exponential, and coalescent exponential population were adopted for the molecular evolutionary analysis of *RdRp*. The lengths of the Bayesian MCMC chains and samples are listed in Table S2. Effective sample sizes (ESS) were calculated using Tracer and the convergence of all parameters was confirmed if the ESS was greater than 200. After a 10% burn-in, phylogenetic trees were generated using TreeAnnotator (v.2.6.7) and rendered using FigTree (v.1.4.0). Representative strains of each cluster were selected based on the most recent age of detection within the cluster. In addition, to compare the amino acid sequences of these representative strains, the GII.6 strain (AB039777) prototype was determined based on a previous report [10]. Molecular evolutionary rates were estimated using suitable models selected for each dataset, as described above. Statistical analyses were performed using the Kruskal–Wallis *t*-test for EZR [30].

### 2.3. Phylogenetic Distance Analyses

To calculate the phylogenetic distances among the strains, we used MEGA7 software [31]. The best substitution models were estimated using the jModelTest2 program. The phylogenetic distances between the present GII.6 strains were calculated from the pairwise maximum likelihood (ML) tree of the ML tree using the Patristic program [32].

### 2.4. Phylodynamic Analyses

To assess the phylodynamics of the GII.6 strains, the effective population sizes of the *RdRp* region and *VP1* gene were calculated using Bayesian skyline plot (BSP) analysis implemented in the BEAST package [27]. Similar to the Bayesian MCMC method, the best substitution and clock models were selected. A Bayesian skyline plot and the 95% highest probability density (HPD) were visualized using Tracer [33].

### 2.5. Selective Pressure Analyses

The non-synonymous (*dN*) and synonymous (*dS*) substitution rates at each amino acid site were calculated to identify the selective pressure sites for the *RdRp* region and *VP1* gene using the Datamonkey server (https://www.datamonkey.org/ accessed on 8 October 2022) [34]. Five algorithms—single likelihood ancestor counting (SLAC), fixed-effects likelihood (FEL), internal fixed-effects likelihood (IFEL), the mixed-effects model of evolution (MEME) method, and the fast, unconstrained Bayesian approximation (FUBAR) method—were used to identify positively selected sites, and all of them except FUBAR were used to detect negatively selected sites. The significance level was set at *p* < 0.05 for SLAC, FEL, IFEL, and MEME. Evidence of selective pressure for FUBAR was supported by a posterior probability > 0.9. In the positive selection analysis, sites common to more than four methods were regarded as positive selection sites, whereas in the negative selection analysis, sites common to more than three methods were considered negative selection sites.

### 2.6. Construction of the 3D Structure of RdRp and VP1 Proteins

To compare the VP1 and RdRp protein structures among genotypes, three-dimensional (3D) structural models of VP1 and RdRp proteins were constructed for each genotype using homology modeling. First, 3D structural models of VP1 in representative strains of each genotype (AB039777, LC122916, MH791993, MK956199, and JX989075) were generated using Protein Data Bank (PDB) ID: 6OTF as a template. Then, five models for each *VP1* genotype were generated using Modellar software (version 9.23) [35]. These models were evaluated by Ramachandran plot analysis using WinCoot implemented in the CCP4 package [36] and the best-scoring models were chosen. Finally, the energy of the selected models for each strain was minimized using GROMOS96 implemented in the Swiss PDB viewer (ver4.1.0) [37]. Using a similar procedure, the models of RdRp protein in each representative strain (AB039777, LC122916, LC760173, MK956199, and JX989075) (Table S1) were constructed using the crystal structure of RdRp (PDB ID:1SH0) as a template.

### 2.7. Conformational B-Cell Epitope Prediction

PDB files of the crystal structures of the GII.2 VP1 protein (PDB ID:6OTF) and FASTA files of their amino acid sequences were downloaded from PDB (https://pdbj.org/?lang=ja accessed on 30 August 2022) to use as templates in the homology modeling method. To assess the conformational B-cell epitopes of the constructed VP1 protein models, four methods, DiscoTope 2.0 [38], ElliPro [39], SEMA [40], and SEPPA [41], were used, with cutoff values of −3.7, 0.5, 0.76, and 0.064, respectively. Regions with amino acid sequences predicted by three or more of these methods and those contiguous with three or more residues were regarded as conformational epitopes. Furthermore, conformational epitopes were mapped onto the VP1 protein models constructed above.

## 3. Results

### 3.1. Time-Scaled Phylogeny of the RdRp Region and VP1 Gene in HuNoV GII.P6-GII.6 and GII.P7-GII.6

**Figure 1:**
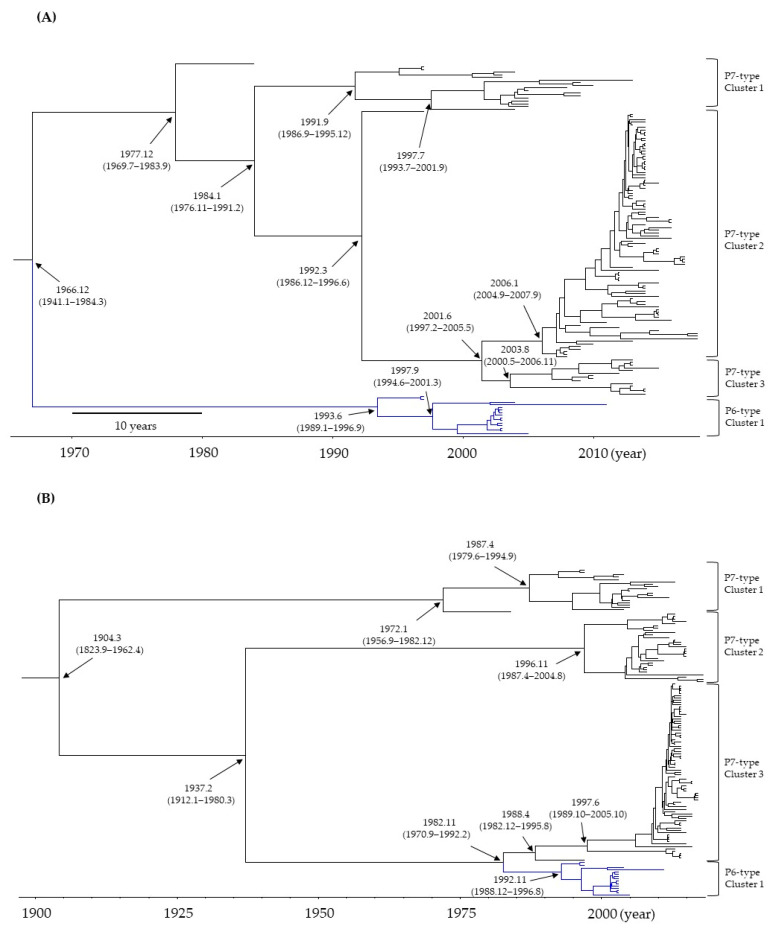
Time-scaled phylogenetic tree of the (**A**) *RdRp* region and (**B**) *VP1* gene in GII.P6-GII.6 (15 strains) and GII.P7-GII.6 strains (126 strains) of the human norovirus (HuNoV) constructed using the Bayesian MCMC method. The divergence times with 95% highest probability densities (HPDs) are indicated on the phylogenetic tree.

Time-scale phylogenetic trees were constructed based on the full-length nucleotides of the *RdRp* region and *VP1* gene using the Bayesian MCMC method. First, as shown in Figure 1A, a common ancestor of the P6- and P7-type *RdRp* regions diverged around December 1966 (mean; 95% HPDs, January 1941–March 1984). Subsequently, the P6- or P7-type *RdRp* regions further diverged and formed clusters 1 and 3, respectively. The main divergence times are shown in Figure 1A. The results suggested that a common ancestor of the P6- and P7-type *RdRp* region diverged approximately 50 years ago and evolved. 

Next, as shown in Figure 1B, a common ancestor of the GII.6 *VP1* gene diverged around March 1904 (mean; 95% HPDs, September 1823–April 1962). Thereafter, the genes diverged to form four clusters. The main divergence times are shown in Figure 1B. Finally, the GII.6 strains with the P6-type formed only one cluster, while the GII.6 strains with the P7-type formed three independent clusters. Furthermore, this phylogenetic tree estimated that a common ancestor of the P6- and P7-type *VP1* genes diverged around November 1982 (mean; 95% HPDs, September 1970–February 1992). Thus, this time may be estimated as a recombination event between the GII.P6-GII.6 and GII.P7-GII.6 genomes in the present strains. 

### 3.2. Evolutionary Rates of the RdRp Region and VP1 Gene in HuNoV GII.P6-GII.6 and GII.P7-GII.6

We also calculated the evolutionary rates using the Bayesian MCMC method. As shown in Table 1, the evolutionary rate was higher for GII.6 VP1 than the RdRp region, including P6- and P7-types (141 strains). The evolutionary rate was higher for the P6-type RdRp region than the P7-type RdRp. The evolutionary rate was higher for the P6-type GII.6 VP1 than the P7-type GII.6 VP1. These results suggest that the RdRp region and VP1 gene in the present strains evolved independently, and the evolutionary rates were significantly distinct.

**Table 1:** Evolutionary rates of the present GII.6 strains.

Region/Gene	Evolutionary Rates (95% HPDs) (Substitutions/Site/Year)	Compared Groups and Statistical Values
All *RdRp* region (141 strains) P6-type 15 strains; P7-type 126 strains	3.287 × 10^−3^ (2.489 × 10^−3^–4.098 × 10^−3^)	All *RdRp* region vs. All GII.6 *VP1* gene *p* < 0.001
All GII.6 *VP1* gene (141 strains) P6-type 15 strains; P7-type 126 strains	3.345 × 10^−3^ (2.295 × 10^−3^–4.419 × 10^−3^)	
P6-type *RdRp* region (15 strains)	5.063 × 10^−3^ (3.525 × 10^−3^–6.595 × 10^−3^)	P6-type *RdRp* region vs. P7-type *RdRp* region *p* < 0.001
P7-type *RdRp* region (126 strains)	3.022 × 10^−3^ (2.268 × 10^−3^–3.775 × 10^−3^)	
GII.P6-GII.6 *VP1* gene (15 strains)	3.725 × 10^−3^ (1.843 × 10^−3^–5.549 × 10^−3^)	GII.P6-GII.6 *VP1* gene vs. GII. P7-GII.6 *VP1* gene *p* < 0.001
GII.P7-GII.6 *VP1* gene (126 strains)	3.482 × 10^−3^ (2.419 × 10^−3^–4.568 × 10^−3^)	

### 3.3. Phylogenetic Distances among the Present Strains

**Figure 2:**
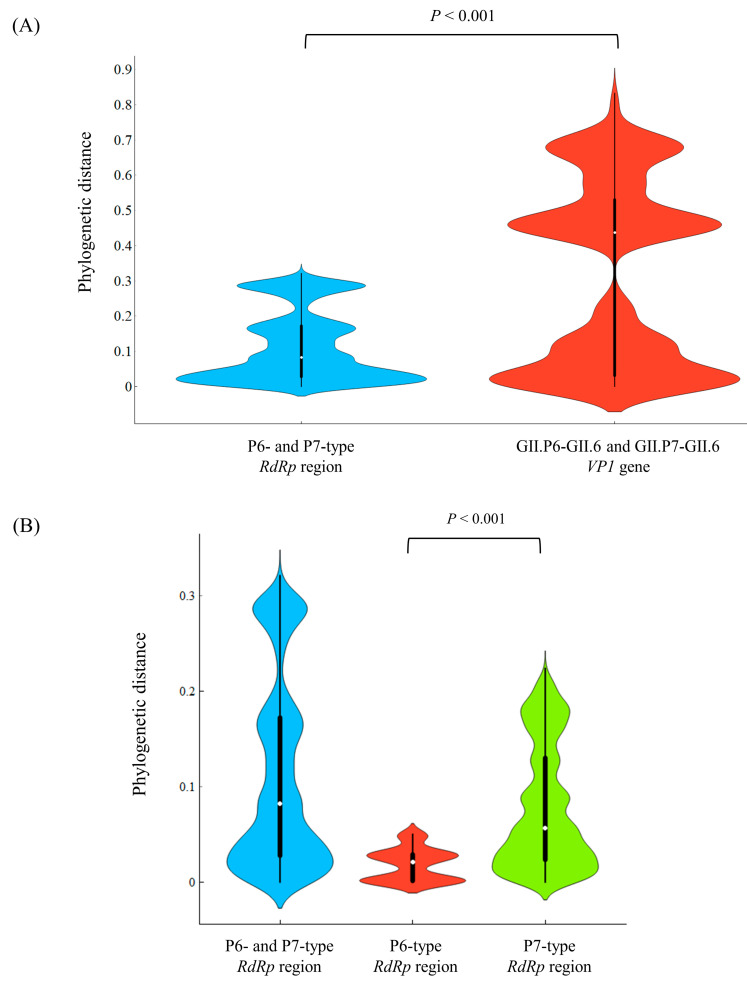

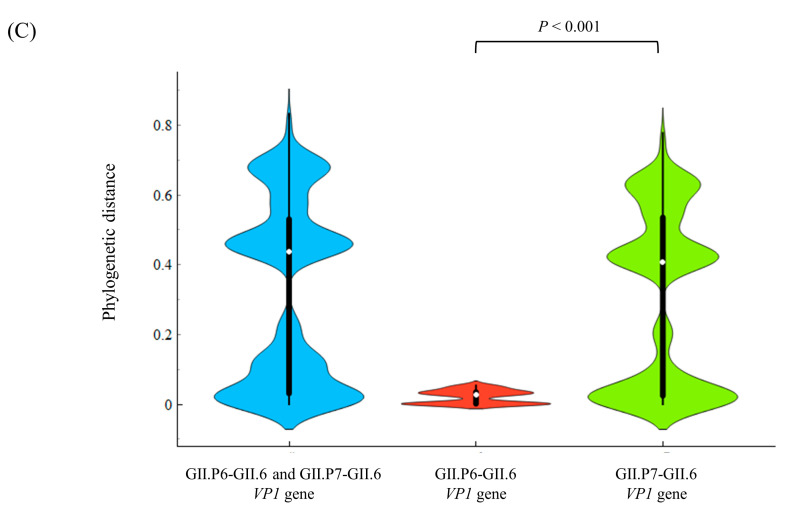
Phylogenetic distances of the (**A**) *RdRp* and *VP1* regions, (**B**) *RdRp* region, and (**C**) *VP1* gene in HuNoV GII.P6-GII.6 and GII.P7-GII.6, represented as violin plots. The width of the violin represents kernel density, indicating the distribution shape of the data. The central thick black bar and thin black line show the interquartile range and all data intervals, respectively. The white dot represents the median.

To assess the genetic divergence of the RdRp region and VP1 gene in the present strains, we calculated their phylogenetic distances. The phylogenetic distances of the P6- and P7-type RdRp regions and the GII.6 VP1 gene were 0.112 ± 0.098 (mean ± 1 standard deviation [SD]) and 0.317 ± 0.259 (mean ± 1 SD). As shown in Figure 2A, the VP1 gene showed statistically greater genetic divergence than the RdRp region (unpaired *t*-test, *p* < 0.001). Moreover, the genetic divergence was greater for the P7-type RdRp region than the P6-type RdRp region (unpaired *t*-test, *p* < 0.001). The detailed statistical data are shown in Table 2.

### 3.4. Phylodynamics of GII.P6-GII.6 and GII.P7-GII.6

**Figure 3:**
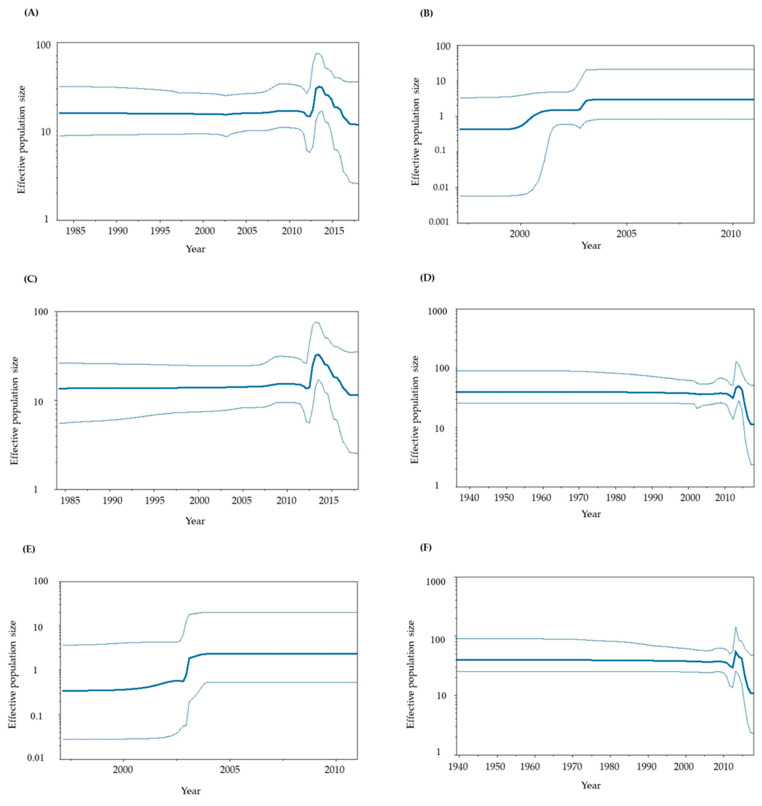
Phylodynamics of the present HuNoV GII.P6-GII.6 and GII.P7-GII.6 strains determined using Bayesian skyline plot analysis. (**A**) Phylodynamics of the P6-type and P7-type *RdRp* regions; (**B**) P6-type *RdRp* region; (**C**) P7-type *RdRp* region; (**D**) P6- and P7-type *VP1* genes; (**E**) P6-type *VP1* gene alone; (**F**) P7-type *VP1* gene alone. The *y*-axis shows the effective population size for each distance, and the *x*-axis represents time (years). The thick line in the centre shows the median effective population sizes, and the thin lines at the top and bottom indicate the 95% HPDs.

To assess the phylodynamics of the present GII.P6-GII.6 and GII.P7-GII.6 strains, we calculated time-scaled genome population sizes using the BSP method (Figure 3A–F). Until approximately 2010, the genome population sizes of both the RdRp region and VP1 gene remained constant. However, significant fluctuations in genome population sizes were observed at around 2010–2018.

### 3.5. Positive Selection Sites in the RdRp and VP1 Proteins

We analysed the positive selection sites in the RdRp and VP1 proteins to estimate selective pressure against the host. No positive selection site was detected in the RdRp protein. In contrast, a few positively selected sites were identified in VP1. Of these, only Lys386His was predicted in the P6-type VP1 protein, whereas Pro354Thr, Pro354Ser, Pro354Gln, Asn390Thr, and Asn390Asp were predicted in the P7-type VP1 protein. These sites were located in the protruding 2 (P2) domain of the protein (Table S4). These results suggest that the GII.P7-GII.6 strains may receive stronger selection pressure from the host than the GII.P6-GII.6 strains. Amino acid substitutions were also observed in sites other than the P2 domain, but no positive selection sites were identified.

### 3.6. Negative Selection Sites in RdRp and VP1 Proteins

In general, negative selection sites may prevent the deterioration of protein function. Therefore, we calculated the number of negative-selection sites in these strains. Many negative selection sites were estimated for the P7-type RdRp protein (205 sites) and P7-type VP1 protein (274 sites). However, a small number of negative selection sites were estimated in the P6-type RdRp protein (3 sites) and P6-type VP1 protein (8 sites) (Table 3). A few irregularly positioned negative selection sites in the P6-type RdRp and VP1 proteins were identified. Details of these negative selection sites are shown in Supplementary Table S3A,B. In both the RdRp and VP1 proteins, amino acid variations in the negative selection sites were often located in non-overlapping positions.

### 3.7. 3D Mapping Relationships between Amino Acid Substitutions and Active Sites of the RdRp Dimer Proteins

**Figure 4:**
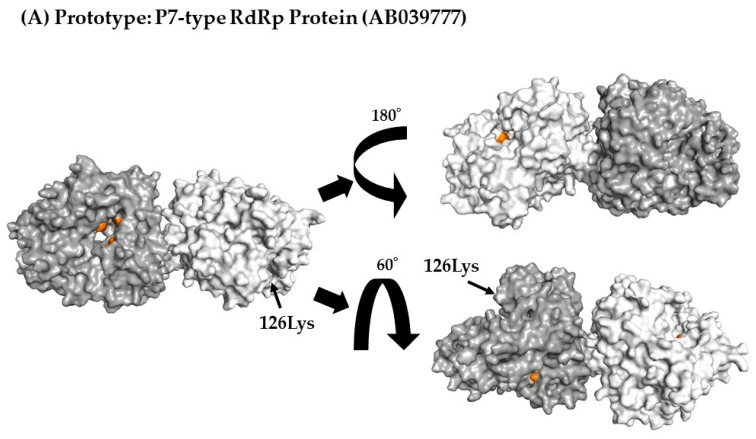

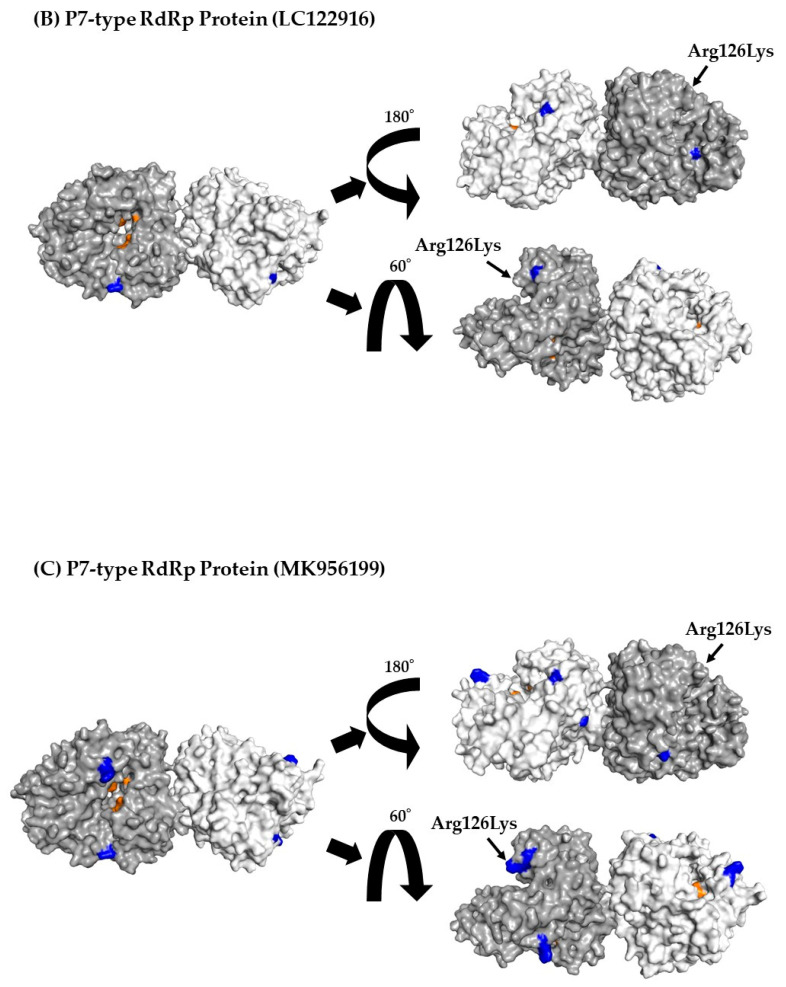

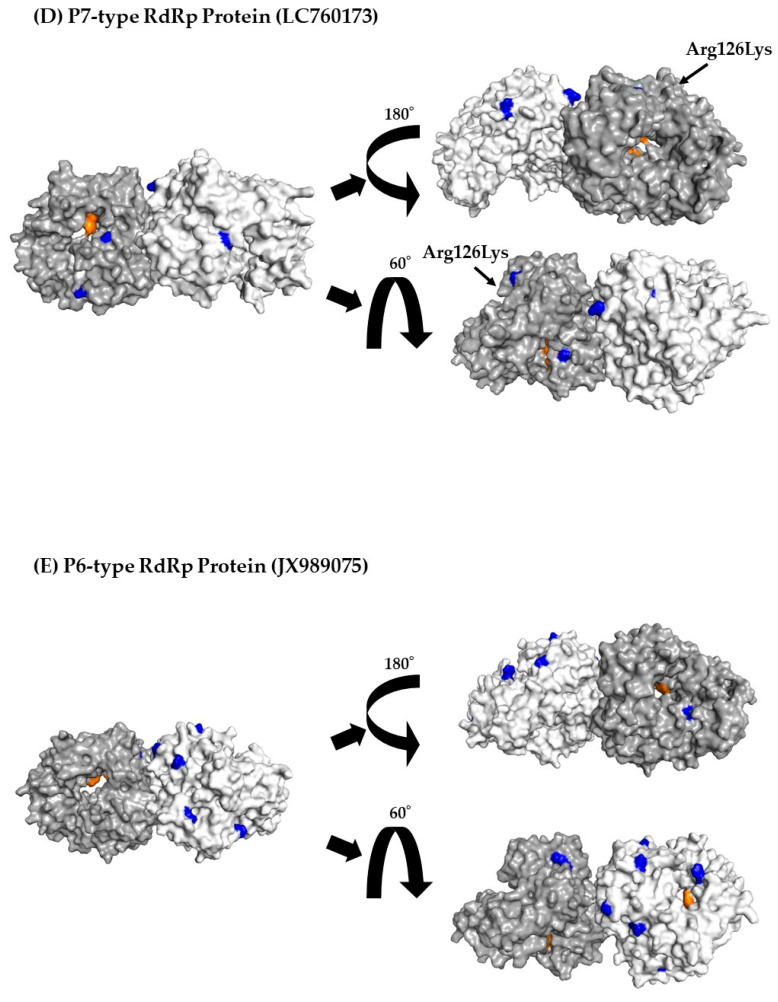
Three-dimensional (3D) RdRp protein (dimer) structure and mapping of amino acid substitutions and active sites. Illustration shows the 3D structures of RdRp protein in the prototype and the most recent strain for each cluster. The strains in each figure are as follows: (**A**) a P7-type prototype strain (AB039777); (**B**) a P7-type strain (LC122916) in cluster 1; (**C**) a P7-type strain (MK956199) in cluster 2; (**D**) a P7-type strain (LC760173) in cluster 3; (**E**) a P6-type strain (JX989075) in cluster 1. The chains of the dimer structure are coloured dark grey (chain A) and light grey (chain B). Amino acid substitutions in each variant strain relative to the prototype strain are shown in blue, and the active sites are shown in orange.

To better assess the relationships between amino acid substitutions and the active sites of RdRp proteins, we constructed 3D RdRp dimers and mapped them. Within RdRp, there were few amino acid substitutions in both the P6 and P7 types, but none were observed in the RdRp active sites (aa182, aa242, aa243, aa300, aa309, aa343, and aa344) (Figure 4A–E).

### 3.8. 3D Mapping of the Positive Selection Sites and Conformational Epitopes in the VP1 Trimer Proteins

**Figure 5:**
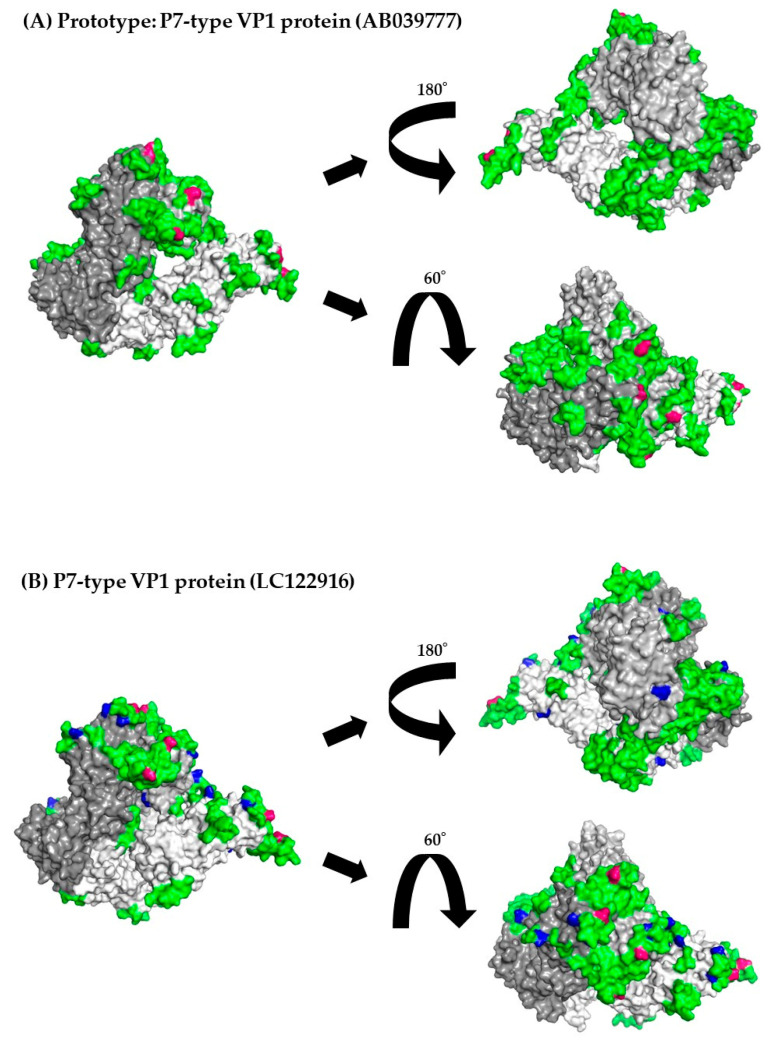

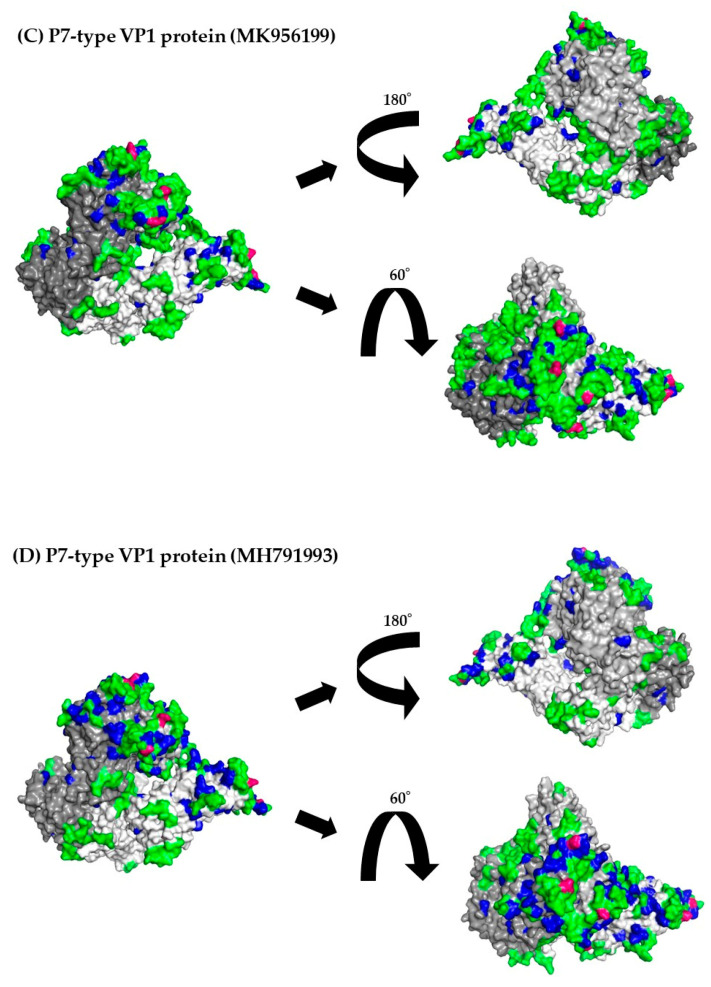

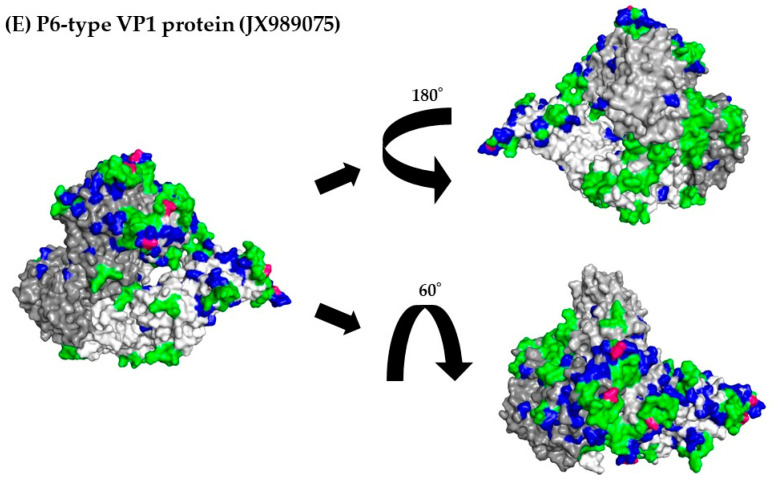
3D mapping of the positive selection sites and conformational epitopes in the VP1 protein (trimer). Illustration shows the 3D structure of the VP1 protein in the prototype and the most recent strain for each cluster. The strains in each figure are as follows: (**A**) a P7-type prototype strain (AB039777); (**B**) a P7-type strain (LC122916) in cluster 1; (**C**) a P7-type strain (MK956199) in cluster 2; (**D**) a P7-type strain (MH791993) in cluster 3; (**E**) a P6-type strain (JX989075) in cluster 1. Chains of the trimeric structures are coloured in dark grey (chain A), light grey (chain B), and white (chain C). Conformational epitopes of each strain are indicated in green. Amino acid substitutions of each strain are indicated in blue. Positive selection sites are coloured red. When amino acid substitutions overlapped with conformational epitopes, the amino acid substitutions were given priority and coloured blue. The amino acid sequences and details are provided in Supplementary Table S4.

Furthermore, to better evaluate the locations of positive selection sites and conformational epitopes on the VP1 protein, we constructed and mapped 3D VP1 trimer proteins. First, as shown in Figure 5A–E and Table 3, and Section 3.5, positive selection sites for both P6- and P7-type VP1 proteins were located in the P2 domain. Of these, the positive selection sites in the P7-type VP1 proteins (Pro354Thr, Pro354Ser, Pro354Gln, Asn390Thr, and Asn390Asp) overlapped with some conformational epitopes (Table 3 and Table S4), whereas a positive selection site (Lys386His) in P6-type VP1 proteins did not. These results suggest that the positive selection sites in P7-type GII.6 VP1 proteins escaped amino acid mutations. In addition, in both representative strains, amino acid mutations overlapped with the conformational epitope at many sites.

**Table 3:** Number of amino acid residues of predicted positive and negative selection sites in HuNoVGII.6.

Region/Gene	Number of Negative Selection Sites	Number of Positive Selection Sites	Estimated as Positive Selective Sites
P6 type and P7 type *RdRp* region	258	0	—
P6 type *RdRp* region	3	0	—
P7 type *RdRp* region	205	1	126Lys, Lys126Arg
GII.P6-GII.6 and GII.P7-GII.6 *VP1* gene	298	2	354Pro, 390Asn
GII.P6-GII.6 *VP1* gene	8	1	Lys386His
GII.P7-GII.6 *VP1* gene	274	2	Pro354Thr, Ser and Gln, Asn390Thr and Asp

## 4. Discussion

To better understand the evolution of HuNoV GII.6 strains with different *RdRp* types (P6 and P7), we analyzed both the *RdRp* region and *VP1* gene using various authentic bioinformatics technologies. First, a time-scaled phylogenetic tree showed that a common ancestor of the P6- and P7-type *RdRp* region emerged approximately 50 years ago and uniquely evolved and formed clusters. A common ancestor of P6- and P7-type GII.6 *VP1* gene emerged approximately 110 years ago and formed clusters. The dominant type for both the *RdRp* region and *VP1* gene was P7-type (Figure 1A,B). Secondly, the evolutionary rates of both the P6-type *RdRp* region and *VP1* gene were faster than those of the P7-type *RdRp* region and *VP1* gene (Table 1). Next, the phylogenetic distances of the P7-type *RdRp* region and *VP1* gene were wider than those of the P6-type *RdRp* region and *VP1* gene. Furthermore, phylodynamic data showed that the *RdRp* region and *VP1* gene population sizes fluctuated after 2000 (Figure 3A–F). Some positive selection sites in the VP1 proteins were estimated, and these were located in the antigenicity-related P2 domain. Among these, the positive selection sites in the P7-type VP1 protein overlapped with the conformational epitopes (Figure 5A–D and Table S4). These data imply that the GII.6 *VP1* gene and VP1 protein uniquely evolved because of recombination between the P6- and P7-type *RdRp* regions in the HuNoV GII.P6-GII.6 and GII.P7-GII.6 genomes.

A previous report regarding the evolutionary analyses of the *RdRp* region of various HuNoV genotypes showed that the P6- and P7-type *RdRp* region diverged from a common ancestor of other *RdRp* genotypes, including P18, P15, and P20 [42]. This report also estimated that the divergence year of the P6- and P7-types of the *RdRp* region was in the 1960s [42]. This may be compatible with the present data (December 1966). Moreover, the topologies of the previous time-scaled evolutionary tree and our tree were similar [42]. Although this and other reports did not show the evolutionary rates of each *RdRp* genotype, the evolutionary rates of various *RdRp* genotypes were estimated as 2.52 × 10^−3^ s/s/y to 3.12 × 10^−3^ s/s/y. The present data are also compatible with the data from a previous report [42]. These results suggested that the P6- and P7-type *RdRp* regions are genetically related. 

Next, the HuNoV *RdRp* region/RdRp protein may have affected the evolution of the *VP1* gene/VP1 protein [22,23]. As shown in Figure 1, the phylogeny of the *VP1* gene in GII.P6-GII.6 and GII.P7-GII.6 was clearly divided and evolved uniquely. In contrast, in the present study, the topology of the evolutionary tree of the *RdRp* region was similar to that of the *VP1* gene. Notably, the phylogeny of ORF1 and ORF2 was topologically similar in other genogroups and genotypes [43]. Previous reports have also suggested that recombination between the HuNoV genome ORF1, incorporating the *RdRp* region and ORF2, incorporating *VP1* gene, affects *VP1* gene/VP1 protein evolution and HuNoV antigenicity [8,22,23]. For example, during the 2016/2017 season, recombination between different lineages of the P16-type *RdRp* region in the GII.P16-GII.2 strains occurred, and the recombinant caused large outbreaks of acute gastroenteritis in various countries [44,45,46]. Moreover, the GII.4 genotype caused a gastroenteritis pandemic between 2006 and 2012 [43,47]. Outbreaks may also be associated with recombination between ORF1 and ORF2 in GII.4 strains [48]. Based on previous and the present results, the prevalence of GII.P7-GII.2 strains was due to the recombination of P6- and P7-type *RdRp* regions. Moreover, the acquisition of new polymerases in recombinant strains may alter the evolution rate of the *VP1* gene [49]. Among Kawasaki 2014 type-detected cases, a norovirus GII.17 variant that was predominant in Hong Kong from 2014 to 2015 was significantly more common than GII.4 in elderly cases. GII.17 VP1 protein evolution is estimated to be faster (by an order of magnitude) than *VP1* gene evolution in GII.4 [50]. This suggests that recombination may alter the susceptibility and evolutionary rate of the VP1 protein in GII.17. Although we did not analyse VP1 functional changes pre- and post-recombination between GII.P6 and GII.P7 in this study, a similar effect may have occurred in the GII.P7-GII.6 and GII.P6-GII.6 strains. Furthermore, the evolutionary rates of the *VP1* gene combined with P6- and P7-type *RdRp* regions were estimated as 5.063 × 10^−3^ s/s/y and 3.022 × 10^−3^ s/s/y, respectively. Previous data estimated the mean rates of various GII.2 genotype strains (GII.1 to GII.22) as 3.21 × 10^−3^ to 4.30 × 10^−3^ s/s/y [51]. Thus, these values and the present data may be similar [52]. Taken together, these findings provide information on the evolutionary history of these viral strains and suggest that recombination events may have played a pivotal role in their evolution.

We estimated the genetic divergence of the P6- and P7-type *RdRp* regions and P6- and P7-type *VP1* genes in the present strains. First, a larger divergence of P7-type *RdRp* regions and P7-type *VP1* genes was estimated compared to that of the P6-type *VP1* gene. In the present study, the number of P6-type strains was relatively small (15 strains), although statistical analyses were performed.

We also analyzed the phylodynamics of the *RdRp* region and *VP1* gene. The results showed that the genome population size of GII.P6-GII.6 increased around 2000–2003, while the genome population size of GII.P7-GII.6 increased after 2010 and peaked around 2014. Previous epidemiological studies conducted in Shanghai, China and East Java, Indonesia showed that the detection of the P7-type in HuNoV cases peaked in 2014–2015, although the sample size was small [53,54]. Moreover, another molecular epidemiological study suggested that GII.6 had a biphasic prevalence between 2000 and 2005 and 2007 and 2010. Thus, the present phylodynamic data may reflect the prevalence of GII.P6-GII.6 and GII.P7-GII.6, although we could not determine the factors underlying these changes.

To evaluate the functional and evolutionary characteristics of the P6- and P7-type RdRp proteins, we constructed 3D dimeric RdRp proteins and mapped them with amino acid substitutions (Figure 4). Several amino acid substitutions were also identified. Previous reports have suggested that some amino acid substitutions are associated with replication efficacy [8,48]. For example, the efficacy of HuNoV genome replication is increased by amino acid substitutions (291Thr or 291Val) in various RdRp proteins [52]. However, no substitutions in the active sites were found in the P6- and P7-type RdRp proteins. Furthermore, both GII.P6-GII.6 and GII.P7-GII.6 RdRp proteins had a relatively small number of amino acid substitutions and no identifiable positive selection site. These results are compatible with previous reports investigating other genogroups (GI) and genotypes [42,55]. This is partly because RdRp, which is not a target of neutralising antibodies, undergoes less selective pressure from host immune systems than VP1.

We also constructed P6- and P7-type 3D trimeric VP1 proteins (Figure 5). Previous reports have shown that the P2 domain may act not only as a host cell-binding site, but also as a major part of the HuNoV antigen [56,57]. Therefore, amino acid substitutions in this domain may be associated with infectivity and antigenicity [56,57]. Moreover, positively selected sites may function as escape mutations in the host [58]. In the present study, some conformational epitopes were identified in both RdRp-type VP1 proteins. Some of these were located in the P2 domain. Positively selected sites were also identified. Moreover, the amino acid positions of the conformational epitopes and positive selections between the P6- and P7-type VP1 proteins were distinct. These results suggested that the P6- and P7-type VP1 proteins have distinct antigenicity, and both may undergo distinct selective pressure from host defence systems (i.e., host immunity). To our knowledge, the present study is the first to analyse the differences in antigenicity between GII.P7-GII.6 and GII.P6-GII.6, although we did not examine this in vitro.

Next, we evaluated negative selection sites for the RdRp and VP1 proteins (Table 3 and Table S3). Many negative selection sites in P7-type RdRp (205 sites) and VP1 proteins (274 sites) were estimated, while P6-type RdRp and VP1 proteins were small. In general, negative selection sites play a role in preventing the deterioration of viral protein function [46]. Thus, the present negative selection data may indicate the maintenance of RdRp and VP1 protein function. Furthermore, a small number of negative selection sites in P6-type RdRp and VP1 proteins were estimated. This may be because of the relatively small number of strains used in this study (15 strains). 

The present study has some limitations. First, it lacks in vitro and in vivo approaches. Bioinformatics techniques are crucial in molecular evolution analyses, but they do not always reflect actual evolutionary trajectories. Our antigenicity analyses should be validated using in vitro and in vivo approaches in the future. Second, the study included a relatively small number of GII.P6-GII.6 strains. Despite collecting additional HuNoV GII.6 data from each local government public health institution, the number of P6- and P7-type *RdRp* strains that we collected and analysed was 15 and 126, respectively. Therefore, our results regarding the P6-type may be biased. Considering this limitation, further evolutionary analyses should be conducted after additional P6-type strains are detected.

## 5. Conclusions

In this study, to better understand the evolution of the HuNoV GII.P6-GII.6 and GII.P7-GII.6 strains, we performed a detailed analysis of both the *RdRp* region and *VP1* gene in these viruses using various bioinformatics methods. A common ancestor of the P6- and P7-type *RdRp* region emerged approximately 50 years ago and formed clusters. A common ancestor of the P6- and P7-type *VP1* gene emerged approximately 110 years ago. Moreover, both *RdRp* region and *VP1* gene have evolved uniquely. The evolutionary rates of the P6-type *RdRp* region and P6-type *VP1* gene were faster than the evolutionary rates of the P7-type *RdRp* region and *VP1* genes. More genetic divergence was observed in the P7-type *RdRp* region and *VP1* gene than in the P6-type *RdRp* region and *VP1* gene. The phylodynamics of the *RdRp* region and *VP1* fluctuated after 2000. Some positive selection sites in the VP1 proteins were located in the antigenicity-related P2 domain, and these sites in the P7-type VP1 protein overlapped with the conformational epitopes. Taken together, the GII.6 *VP1* and VP1 proteins evolved uniquely due to recombination between the P6- and P7-type *RdRp* regions in the HuNoV GII.P6-GII.6 and GII.P7-GII.6 strains.

## Figures and Tables

**Table 2:** Phylogenetic distance of the present strains.

Region/Gene	Phylogenetic Distance (Mean ± SD)	Phylogenetic Distance (Median [IQR])
All *RdRp* region (141 strains)	0.112 ± 0.098	0.082 (0.028–0.172)
All GII.6 *VP1* gene (141 strains)	0.317 ± 0.259	0.437 (0.031–0.530)
P6-type *RdRp* region (15 strains)	0.018 ± 0.017	0.021 (0.001–0.029)
P7-type *RdRp* region (126 strains)	0.078 ± 0.064	0.057 (0.023–0.130)
GII.P6-GII.6 *VP1* gene (15 strains)	0.021 ± 0.019	0.027 (0.001–0.035)
GII.P7-GII.6 *VP1* gene (126 strains)	0.305 ± 0.249	0.408 (0.024–0.535)

## Data Availability

The datasets generated and/or analyzed in the present study are available from the corresponding author upon reasonable request.

## Supplementary Materials

The following supporting information can be downloaded from: https://www.mdpi.com/article/10.3390/v15071497/s1, Table S1. List of GII.P6-GII.6 and GII.P7-GII.6 strains used in this study. Table S2. Parameters used in Bayesian Markov chain Monte Carlo (MCMC) analyses. Table S3a. Details of P6- and P7-type *RdRp* region negative selection sites in comparing with each genotype. Table S3b. Details of GII.P6-GII.6 and GII.P7-GII.6 *VP1* gene negative selection sites in comparison with each genotype. Table S4. Detailed amino acid sequences of the *VP1* gene for representative and prototype strains of each cluster used in this study.
