# Supplementary material for: Molecular Evolutionary Analyses of the RNA-Dependent RNA Polymerase (RdRp) Region and VP1 Gene in Human Norovirus Genotypes GII.P6-GII.6 and GII.P7-GII.6
Source: Viruses. 2023 Jul 1;15(7):1497. doi: 10.3390/v15071497 (PMC10383674; doi:10.3390/v15071497)
Supplement: Supplementary file 1 [file viruses-15-01497-s001.zip › Table S1. List of GII.P6-GII.6 and GII.P7-GII.6 strains used in this study._230521.pdf.zip!/Table S1. List of GII.P6-GII.6 and GII.P7-GII.6 strains used in this study._230521.pdf]

Table S1. List of GII.P6-GII.6 and GII.P7-GII.6 strains used in this study.

GenBank Accesion No.	strain	Isolated/collected year	Country/region	polymerase type
JX846927	JX846927.1_Norovirus_HuGII.6CH	1984	USA	GII.P7
AB039778	AB039778.1_Norwalk-like_virus_	1997	Japan/ Saitama U16	GII.P6
AB039779	AB039779.1_Norwalk-like_virus_	1997	Japan/ Saitama U17	GII.P6
AB039776	AB039776.1_Norwalk-like_virus_	1997	Japan/ Saitama U3	GII.P7
AB039777	AB039777.1_Norwalk-like_virus_	1997	Japan/ Saitama U4	GII.P7
GU930737	GU930737.1_Norwalk-like_virus_	1997	USA: Texas	GII.P7
LC122923	GII.6-GII.P6HI-1Nov2004_2004	2004	Japan/ Hiroshima	GII.P6
LC122924	GII.6-GII.P6TO-59Nov2003_2003	2003	Japan/ Tochigi	GII.P6
LC122928	GII.6-GII.P6TO-60Sep2003_2003	2003	Japan/ Tochigi	GII.P6
LC122925	GII.6-GII.P6TO-61Oct2003_2003	2003	Japan/ Tochigi	GII.P6
LC122933	GII.6-GII.P6TO-62Oct2003_2003	2003	Japan/ Tochigi	GII.P6
LC122926	GII.6-GII.P6TO-63Oct2003_2003	2003	Japan/ Tochigi	GII.P6
LC122931	GII.6-GII.P6TO-64Oct2003_2003	2003	Japan/ Tochigi	GII.P6
LC122929	GII.6-GII.P6TO-65Oct2003_2003	2003	Japan/ Tochigi	GII.P6
LC122930	GII.6-GII.P6TO-66Nov2003_2003	2003	Japan/ Tochigi	GII.P6
LC122932	GII.6-GII.P6TO-68Dec2003_2003	2003	Japan/ Tochigi	GII.P6
LC760142	GII.6-GII.P6TO-71Dec2003_2003	2003	Japan/ Tochigi	GII.P6
LC122921	GII.6-GII.P7TO-52Apr2003_2003	2003	Japan/ Tochigi	GII.P7
LC122922	GII.6-GII.P7TO-53Apr2003_2003	2003	Japan/ Tochigi	GII.P7
LC122919	GII.6-GII.P7HO-33Mar2004_2004	2004	Japan/ Hokkaido	GII.P7
LC122920	GII.6-GII.P7HO-34Nov2004_2004	2004	Japan/ Hokkaido	GII.P7
LC122917	GII.6-GII.P7HO-35Dec2005_2005	2005	Japan/ Hokkaido	GII.P7
LC760178	GII.6-GII.P7TO-1022005_2005	2005	Japan/ Tochigi	GII.P7
LC760177	GII.6-GII.P7TO-1072005_2005	2005	Japan/ Tochigi	GII.P7
LC760176	GII.6-GII.P7TO-1152005_2005	2005	Japan/ Tochigi	GII.P7
LC122918	GII.6-GII.P7YA-017Feb2005_2005	2005	Japan/ Yamaguchi	GII.P7
LC760179	GII.6-GII.P7HI-142008_2008	2008	Japan/ Hiroshima	GII.P7
LC122893	GII.6-GII.P7HO-37Dec2008_2008	2008	Japan/ Hokkaido	GII.P7
LC122894	GII.6-GII.P7SA-74Dec2008_2008	2008	Japan/ Saitama	GII.P7
LC122895	GII.6-GII.P7SA-75Dec2008_2008	2008	Japan/ Saitama	GII.P7
LC760144	GII.6-GII.P7EH-532009_2009	2009	Japan/ Ehime	GII.P7
LC122892	GII.6-GII.P7EH-54June2009_2009	2009	Japan/ Ehime	GII.P7
LC122896	GII.6-GII.P7HO-38Oct2009_2009	2009	Japan/ Hokkaido	GII.P7
LC760174	GII.6-GII.P7KA-562009_2009	2009	Japan/ Kanagawa	GII.P7

GenBank Accesion No.	strain	Isolated/collected year	Country/region	polymerase type
KM198498	KM198498.1_Norovirus_HuGII2008	2009	Viet Nam	GII.P7
KM198531	KM198531.1_Norovirus_HuGII3011	2009	Viet Nam	GII.P7
KX752057	KX752057.1_Norovirus_GIIHuChin	2009	China	GII.P7
KJ407072	KJ407072.2_Norovirus_HuGII.6HS	2010	USA	GII.P7
KM198530	KM198530.1_Norovirus_HuGII2048	2010	Viet Nam	GII.P7
KM198534	KM198534.1_Norovirus_HuGII3044	2010	Viet Nam	GII.P7
KM198549	KM198549.1_Norovirus_HuGII3047	2010	Viet Nam	GII.P7
LC122897	GII.6-GII.P7YA-5Nov2011_2011	2011	Japan/ Yamaguchi	GII.P7
LC760145	GII.6-GII.P7KA-52012_2012	2012	Japan/ Kanagawa	GII.P7
LC760175	GII.6-GII.P7SA-1382012_2012	2012	Japan/ Saitama	GII.P7
KY424341	KY424341.1_Norovirus_GII.6_iso	2012	USA	GII.P7
KY424342	KY424342.1_Norovirus_GII.6_iso	2012	USA	GII.P7
KY424343	KY424343.1_Norovirus_GII.6_iso	2012	USA	GII.P7
KY424344	KY424344.1_Norovirus_GII.6_iso	2012	USA	GII.P7
LC122891	GII.6-GII.7MI-27Jul2013_2013	2013	Japan/ Miyagi	GII.P7
LC122901	GII.6-GII.7MI-28Dec2013_2013	2013	Japan/ Miyagi	GII.P7
LC760143	GII.6-GII.P7EH-172013_2013	2013	Japan/ Ehime	GII.P7
LC122916	GII.6-GII.P7EH-55Jan2013_2013	2013	Japan/ Ehime	GII.P7
LC122915	GII.6-GII.P7HI-28Nov2013_2013	2013	Japan/ Hiroshima	GII.P7
LC122898	GII.6-GII.P7HI-29Dec2013_2013	2013	Japan/ Hiroshima	GII.P7
LC760146	GII.6-GII.P7KA-572013_2013	2013	Japan/ Kanagawa	GII.P7
LC760147	GII.6-GII.P7KA-582013_2013	2013	Japan/ Kanagawa	GII.P7
LC760171	GII.6-GII.P7KA-62013_2013	2013	Japan/ Kanagawa	GII.P7
LC760172	GII.6-GII.P7KA-72013_2013	2013	Japan/ Kanagawa	GII.P7
LC760157	GII.6-GII.P7OS-1082013_2013	2013	Japan	GII.P7
LC760158	GII.6-GII.P7OS-1102013_2013	2013	Japan	GII.P7
LC760165	GII.6-GII.P7SA-1402013_2013	2013	Japan/ Saitama	GII.P7
LC760168	GII.6-GII.P7YA-122013_2013	2013	Japan/ Yamaguchi	GII.P7
KM267740	KM267740.1_Norovirus_GII_isola	2013	Taiwan	GII.P7
KM267742	KM267742.1_Norovirus_GII_isola	2013	Taiwan	GII.P7
KM267743	KM267743.1_Norovirus_GII_isola	2013	Taiwan	GII.P7
MF140676	MF140676.1_Norovirus_GII_strai	2013	Netherlands	GII.P7
MF140677	MF140677.1_Norovirus_GII_strai	2013	Netherlands	GII.P7
LC122908	GII.6-GII.7MI-29Jan2014_2014	2014	Japan/ Miyagi	GII.P7
LC122909	GII.6-GII.P7EH-18Jan2014_2014	2014	Japan/ Ehime	GII.P7

GenBank Accesion No.	strain	Isolated/collected year	Country/region	polymerase type
LC122910	GII.6-GII.P7EH-19Apr2014_2014	2014	Japan/ Ehime	GII.P7
LC122900	GII.6-GII.P7HI-33Mar2014_2014	2014	Japan/ Hiroshima	GII.P7
LC122899	GII.6-GII.P7HI-34Mar2014_2014	2014	Japan/ Hiroshima	GII.P7
LC122903	GII.6-GII.P7HI-35Apr2014_2014	2014	Japan/ Hiroshima	GII.P7
LC122912	GII.6-GII.P7HI-36Apr2014_2014	2014	Japan/ Hiroshima	GII.P7
LC122904	GII.6-GII.P7HI-37May2014_2014	2014	Japan/ Hiroshima	GII.P7
LC122905	GII.6-GII.P7HI-38May2014_2014	2014	Japan/ Hiroshima	GII.P7
LC122906	GII.6-GII.P7HI-39Jun2014_2014	2014	Japan/ Hiroshima	GII.P7
LC122911	GII.6-GII.P7HO-39Jun2014_2014	2014	Japan/ Hokkaido	GII.P7
LC122902	GII.6-GII.P7KA-16Jan2014_2014	2014	Japan/ Kanagawa	GII.P7
LC122907	GII.6-GII.P7KA-17Feb2014_2014	2014	Japan/ Kanagawa	GII.P7
LC760148	GII.6-GII.P7KA-592014_2014	2014	Japan/ Kanagawa	GII.P7
LC760149	GII.6-GII.P7KA-812014_2014	2014	Japan/ Kanagawa	GII.P7
LC760150	GII.6-GII.P7MI-672014_2014	2014	Japan/ Miyagi	GII.P7
LC670151	GII.6-GII.P7MI-682014_2014	2014	Japan/ Miyagi	GII.P7
HQ169542	HQ169542.2_Norovirus_HuGII.618	2005	USA	GII.P6
LC670152	GII.6-GII.P7MI-692014_2014	2014	Japan/ Miyagi	GII.P7
JX989075	JX989075.1_Norovirus_HuGII.6GZ	2011	China	GII.P6
LC670153	GII.6-GII.P7MI-702014_2014	2014	Japan/ Miyagi	GII.P7
LC670154	GII.6-GII.P7MI-712014_2014	2014	Japan/ Miyagi	GII.P7
LC670155	GII.6-GII.P7MI-732014_2014	2014	Japan/ Miyagi	GII.P7
LC670156	GII.6-GII.P7MI-742014_2014	2014	Japan/ Miyagi	GII.P7
LC670159	GII.6-GII.P7OS-1112014_2014	2014	Japan	GII.P7
LC670160	GII.6-GII.P7OS-1132014_2014	2014	Japan	GII.P7
LC670161	GII.6-GII.P7OS-1142014_2014	2014	Japan	GII.P7
LC670162	GII.6-GII.P7OS-1322014_2014	2014	Japan	GII.P7
LC670163	GII.6-GII.P7OS-2282014_2014	2014	Japan	GII.P7
LC670164	GII.6-GII.P7OS-2482014_2014	2014	Japan	GII.P7
LC760166	GII.6-GII.P7SA-1412014_2014	2014	Japan/ Saitama	GII.P7
LC122914	GII.6-GII.P7SH-5Jan2014_2014	2014	Japan/ Shizuoka	GII.P7
LC122913	GII.6-GII.P7SH-6Feb2014_2014	2014	Japan/ Shizuoka	GII.P7
LC760169	GII.6-GII.P7TO-1252014_2014	2014	Japan/ Tochigi	GII.P7
LC760170	GII.6-GII.P7TO-1272014_2014	2014	Japan/ Tochigi	GII.P7
KM036373	KM036373.1_Norovirus_HuGII.P7G	2014	Taiwan	GII.P7
KX268709	KX268709.1_Norovirus_HuGII.P7_	2014	USA	GII.P7

GenBank Accesion No.	strain	Isolated/collected year	Country/region	polymerase type
MF140678	MF140678.1_Norovirus_GII_strai	2014	Netherlands	GII.P7
MF140679	MF140679.1_Norovirus_GII_strai	2014	Netherlands	GII.P7
MF140680	MF140680.1_Norovirus_GII_strai	2014	Netherlands	GII.P7
MH218639	MH218639.1_Norovirus_GII_isola	2014	United Kingdom	GII.P7
MH702288	MH702288.1_Norovirus_GII_strai	2014	Bhutan	GII.P7
MK907786	MK907786.1_Norovirus_GII_isola	2014	France/ Nantes	GII.P7
MK907789	MK907789.1_Norovirus_GII_isola	2014	France/ Nantes	GII.P7
LC760173	GII.6-GII.P7MI-752015_2015	2015	Japan/ Miyagi	GII.P7
LC760167	GII.6-GII.P7SA-1422015_2015	2015	Japan/ Saitama	GII.P7
KU935739	KU935739.1_Norovirus_GII_isola	2015	China	GII.P7
KX158281	KX158281.1_Norovirus_GII_strai	2015	Canada	GII.P7
MG571778	MG571778.1_Norovirus_GII.6_clo	2015	Venezuela	GII.P7
MH218640	MH218640.1_Norovirus_GII_isola	2015	United Kingdom	GII.P7
MH218641	MH218641.1_Norovirus_GII_isola	2015	United Kingdom	GII.P7
MH218642	MH218642.1_Norovirus_GII_isola	2015	United Kingdom	GII.P7
MH218643	MH218643.1_Norovirus_GII_isola	2015	United Kingdom	GII.P7
MH218644	MH218644.1_Norovirus_GII_isola	2015	United Kingdom	GII.P7
MH218645	MH218645.1_Norovirus_GII_isola	2015	United Kingdom	GII.P7
MH218650	MH218650.1_Norovirus_GII_isola	2015	United Kingdom	GII.P7
MH218666	MH218666.1_Norovirus_GII_isola	2015	United Kingdom	GII.P7
MH218667	MH218667.1_Norovirus_GII_isola	2015	United Kingdom	GII.P7
MH218673	MH218673.1_Norovirus_GII_isola	2015	United Kingdom	GII.P7
MH218719	MH218719.1_Norovirus_GII_isola	2015	United Kingdom	GII.P7
MN248514	MN248514.1_Norovirus_GII_isola	2015	Belgium	GII.P7
NC_040876	NC_040876.1_Norovirus_GII.P7_G	2015	United Kingdom	GII.P7
MF802550	MF802550.1_Norovirus_GII_isola	2016	China/ Nanjing	GII.P7
MG557654	MG557654.1_Norovirus_GII_isola	2016	Ethiopia	GII.P7
MH218687	MH218687.1_Norovirus_GII_isola	2016	United Kingdom	GII.P7
MH702284	MH702284.1_Norovirus_GII_strai	2016	Cambodia	GII.P7
MH702286	MH702286.1_Norovirus_GII_strai	2016	Cambodia	GII.P7
MH260487	MH260487.1_Norovirus_GII_isola	2017	USA	GII.P7
MH260510	MH260510.1_Norovirus_GII_isola	2017	USA	GII.P7
MH279824	MH279824.1_Norovirus_GII_strai	2017	USA	GII.P7
MH791993	MH791993.1_Norovirus_GII_isola	2017	USA	GII.P7
MK301293	MK301293.1_Norovirus_GII_isola	2018	USA	GII.P7

GenBank Accesion No.	strain	Isolated/collected year	Country/region	polymerase type
MK956198	MK956198.1_Norovirus_GII_isola	2018	France/ Nantes	GII.P7
MK956199	MK956199.1_Norovirus_GII_isola	2018	France/ Nantes	GII.P7
