# Supplementary material for: Molecular Evolutionary Analyses of the RNA-Dependent RNA Polymerase (RdRp) Region and VP1 Gene in Human Norovirus Genotypes GII.P6-GII.6 and GII.P7-GII.6
Source: Viruses. 2023 Jul 1;15(7):1497. doi: 10.3390/v15071497 (PMC10383674; doi:10.3390/v15071497)
Supplement: Supplementary file 1 [file viruses-15-01497-s001.zip › Table S2-4. VP1&RdRp_selection.zip!/Table S2. Parameters used in the Bayesian Marcov chain Monte Carlo (MCMC) analyses..pdf]

Table S2. Parameters used in the Bayesian Marcov chain Monte Carlo (MCMC) analyses of the present HuNoV GII.6 strains.

	Substitution Models	Ganma shape	Proportion invariant	Clock Models	Demographic Models	Chain Length	Log Every
All *RdRp* regions (141 strains) P6-type 15 strains; P7-type 126 strains	SYM+G	0.2	-	Relaxed Clock Exponential	Coalescent Exponential Population	400,000,000	8,000
P6-type *RdRp* region (15 strains)	TPM2+I	-	0.792	Relaxed Clock Exponential	Coalescent Exponential Population	6,000,000,000	40,000
P7-type *RdRp* region (126 strains)	TPM2+G	0.242	0.082	Relaxed Clock Exponential	Coalescent Constant Population	400,000,000	8,000
ALL GII.6 *VP1* genes (141 strains) P6-type 15 strains; P7-type 126 strains	SYM+I+G	1.358	0.524	Relaxed Clock Exponential	Coalescent Exponential Population	400,000,000	8,000
GII.P6-GII.6 *VP1* gene (15 strains)	SYM+G	0.025	-	Relaxed Clock Exponential	Coalescent Constant Population	400,000,000	8,000
GII. P7-GII.6 *VP1* gene (126 strains)	SYM+I+G	1.334	0.518	Relaxed Clock Exponential	Coalescent Constant Population	400,000,000	8,000
