# Supplementary material for: Molecular Evolutionary Analyses of the RNA-Dependent RNA Polymerase (RdRp) Region and VP1 Gene in Human Norovirus Genotypes GII.P6-GII.6 and GII.P7-GII.6
Source: Viruses. 2023 Jul 1;15(7):1497. doi: 10.3390/v15071497 (PMC10383674; doi:10.3390/v15071497)
Supplement: Supplementary file 1 [file viruses-15-01497-s001.zip › Table S2-4. VP1&RdRp_selection.zip!/Table S3. VP1&RdRp_selection.pdf]

Table S3a. Details of P6- and P7-type *RdRp* region negative selection sites in comparing with each genotype.

RdRp genotype	Representative strain	The number of amino acid residues
P6-type (15 strains)	JX989075	213Leu, 370Thr, 463Ile (n=3)
P7-type (126 strains)	AB039777 (prototype)	2Asn, 6Gly, 7Thr, 9Cys, 10Gly, 11Ala, 13Ile, 14Leu, 15Gly, 17Gly, 18Lys, 20Pro, 21Lys, 22Leu, 24Thr, 27Lys, 30Arg, 36Leu, 37Pro, 40Thr, 41Tyr, 42Glu, 47Gly, 48Gly, 51Pro, 53Val, 54Glu, 55Lys, 58Ser, 62Val, 64Arg, 65Asp, 67Leu, 68Lys, 69Pro, 70Phe, 72Glu, 74Arg, 75Gly, 76Lys, 79Arg, 85Glu, 86Ala, 92Asn, 93Val, 94Leu, 97Thr, 99Asp, 102Lys, 106Tyr, 107Ser, 110Cys, 111Ala, 114Asp, 115Lys, 117Asp, 118Ser, 120Gly, 121Ser, 128Asn, 129Asp, 130His, 133Gly, 135Ser, 138Gly, 140Leu, 145Ser, 148Asn, 151Tyr, 152Glu, 155Lys, 156His, 165Leu, 167Asp, 168Glu, 169Leu, 170Val, 171Lys, 173Asp, 184Leu, 186Gly, 187Ser, 188Asp, 190Gly, 191Thr, 195Cys, 198Ala, 200Gly, 201Gly, 204Asp, 205Ser, 209Ser, 210Cys, 212Ala, 215Cys, 216Arg, 217Val, 218Gly, 220Asn, 223Glu, 225Gly, 228Ile, 230Asp, 231Lys, 233Ser, 234Lys, 235Tyr, 237Tyr, 238His, 243Tyr, 244Ser, 249Thr, 255Leu, 256Ser, 260Glu, 264Arg, 267Ala, 268Glu, 272Ala, 275Val, 276Ala, 278Asp, 279Leu, 286Asp, 288Gly, 289Asp, 290Phe, 294Val, 297Gly, 300Ser, 302Val, 304Cys, 307Gln, 310Ser, 313His, 315Ile, 316Leu, 317Thr, 319Ser, 324Val, 327Leu, 331Val, 332Val, 335His, 337Cys, 338Phe, 340Phe, 342Gly, 343Asp, 346Ile, 351Ile, 354Asp, 360Gln, 362Leu, 363Arg, 364Glu, 365Tyr, 366Gly, 368Val, 370Thr, 371Arg, 373Asp, 374Lys, 376Glu, 377Gly, 379Leu, 380Val, 381Ile, 384Asn, 386Thr, 392Arg, 394Ser, 397Arg, 398Asp, 405Lys, 406Leu, 407Asp, 408Gln, 411Ile, 413Arg, 415Leu, 419Arg, 420Gly, 425Asn, 429Ser, 431Val, 433His, 439Gln, 440Leu, 443Leu, 444Leu, 446Glu, 447Ala, 451Gly, 452Pro, 457Lys, 458Val, 463Ile, 465Glu, 466Ile, 469Gly, 472Glu, 482Phe, 483Arg, 502Glu (n=205)

	LC122916	2Asn, 6Gly, 7Thr, 9Cys, 10Gly, 11Ala, 13Ile, 14Leu, 15Gly, 17Gly, 18Lys, 20Pro, 21Lys, 22Leu, 24Thr, 27Lys, 30Arg, 36Leu, 37Pro, 40Thr, 41Tyr, 42Glu, 47Gly, 48Gly, 51Pro, 53Val, 54Glu, 55Lys, 58Ser, 62Val, 64Arg, 65Asp, 67Leu, 68Lys, 69Pro, 70Phe, 72Glu, 74Arg, 75Gly, 76Lys, 79Arg, 85Glu, 86Ala, 92Asn, 93Val, 94Leu, 97Thr, 99Asp, 102Lys, 106Tyr, 107Ser, 110Cys, 111Ala, 114Asp, 115Lys, 117Asp, 118Ser, 120Gly, 121Ser, 128Asn, 129Asp, 130His, 133Gly, 135Ser, 138Gly, 140Leu, 145Ser, 148Asn, 151Tyr, 152Glu, 155Lys, 156His, 165Leu, 167Asp, 168Glu, 169Leu, 170Val, 171Lys, 173Asp, 184Leu, 186Gly, 187Ser, 188Asp, 190Gly, 191Thr, 195Cys, 198Ala, 200Gly, 201Gly, 204Asp, 205Ser, 209Ser, 210Cys, 212Ala, 215Cys, 216Arg, 217Val, 218Gly, 220Asn, 223Glu, 225Gly, 228Ile, 230Asp, 231Lys, 233Ser, 234Lys, 235Tyr, 237Tyr, 238His, 243Tyr, 244Ser, 249Thr, 255Leu, 256Ser, 260Glu, 264Arg, 267Ala, 268Glu, 272Ala, 275Val, 276Ala, 278Asp, 279Leu, 286Asp, 288Gly, 289Asp, 290Phe, 294Val, 297Gly, 300Ser, 302Val, 304Cys, 307Gln, 310Ser, 313His, 315Ile, 316Leu, 317Thr, 319Ser, 324Val, 327Leu, 331Val, 332Val, 335His, 337Cys, 338Phe, 340Phe, 342Gly, 343Asp, 346Ile, 351Ile, 354Asp, 360Gln, 362Leu, 363Arg, 364Glu, 365Tyr, 366Gly, 368Val, 370Thr, 371Arg, 373Asp, 374Lys, 376Glu, 377Gly, 379Leu, 380Val, 381Ile, Asn384Asp, 386Thr, 392Arg, 394Ser, 397Arg, 398Asp, 405Lys, 406Leu, 407Asp, 408Gln, 411Ile, 413Arg, 415Leu, 419Arg, 420Gly, 425Asn, 429Ser, 431Val, 433His, 439Gln, 440Leu, 443Leu, 444Leu, 446Glu, 447Ala, 451Gly, 452Pro, 457Lys, 458Val, 463Ile, 465Glu, 466Ile, 469Gly, 472Glu, 482Phe, 483Arg, 502Glu (n=205)

	LC760173	2Asn, 6Gly, 7Thr, 9Cys, 10Gly, 11Ala, 13Ile, 14Leu, 15Gly, 17Gly, 18Lys, 20Pro, 21Lys, 22Leu, 24Thr, 27Lys, 30Arg, 36Leu, 37Pro, 40Thr, 41Tyr, 42Glu, 47Gly, 48Gly, 51Pro, 53Val, 54Glu, 55Lys, 58Ser, 62Val, 64Arg, 65Asp, 67Leu, 68Lys, 69Pro, 70Phe, 72Glu, 74Arg, 75Gly, 76Lys, 79Arg, 85Glu, 86Ala, 92Asn, 93Val, 94Leu, 97Thr, 99Asp, 102Lys, 106Tyr, 107Ser, 110Cys, 111Ala, 114Asp, 115Lys, 117Asp, 118Ser, 120Gly, 121Ser, 128Asn, 129Asp, 130His, 133Gly, 135Ser, 138Gly, 140Leu, 145Ser, 148Asn, 151Tyr, 152Glu, 155Lys, 156His, 165Leu, 167Asp, 168Glu, 169Leu, 170Val, 171Lys, 173Asp, 184Leu, 186Gly, 187Ser, 188Asp, 190Gly, 191Thr, 195Cys, 198Ala, 200Gly, 201Gly, 204Asp, 205Ser, 209Ser, 210Cys, 212Ala, 215Cys, 216Arg, 217Val, 218Gly, 220Asn, 223Glu, 225Gly, 228Ile, 230Asp, 231Lys, 233Ser, 234Lys, 235Tyr, 237Tyr, 238His, 243Tyr, 244Ser, 249Thr, 255Leu, 256Ser, 260Glu, 264Arg, 267Ala, 268Glu, 272Ala, 275Val, 276Ala, 278Asp, 279Leu, 286Asp, 288Gly, 289Asp, 290Phe, 294Val, 297Gly, 300Ser, 302Val, 304Cys, 307Gln, 310Ser, 313His, 315Ile, 316Leu, 317Thr, 319Ser, 324Val, 327Leu, 331Val, 332Val, 335His, 337Cys, 338Phe, 340Phe, 342Gly, 343Asp, 346Ile, 351Ile, 354Asp, 360Gln, 362Leu, 363Arg, 364Glu, 365Tyr, 366Gly, Val368Ala, 370Thr, 371Arg, 373Asp, 374Lys, 376Glu, 377Gly, 379Leu, 380Val, 381Ile, Asn384Asp, 386Thr, 392Arg, 394Ser, 397Arg, 398Asp, 405Lys, 406Leu, 407Asp, 408Gln, 411Ile, 413Arg, 415Leu, 419Arg, 420Gly, 425Asn, 429Ser, 431Val, 433His, 439Gln, 440Leu, 443Leu, 444Leu, 446Glu, 447Ala, 451Gly, 452Pro, 457Lys, 458Val, 463Ile, 465Glu, 466Ile, 469Gly, 472Glu, 482Phe, 483Arg, 502Glu (n=205)

	MK956199	2Asn, 6Gly, 7Thr, 9Cys, 10Gly, 11Ala, 13Ile, 14Leu, 15Gly, 17Gly, 18Lys, 20Pro, 21Lys, 22Leu, 24Thr, 27Lys, 30Arg, 36Leu, 37Pro, 40Thr, 41Tyr, 42Glu, 47Gly, 48Gly, 51Pro, 53Val, 54Glu, 55Lys, 58Ser, 62Val, 64Arg, 65Asp, 67Leu, 68Lys, 69Pro, 70Phe, 72Glu, 74Arg, 75Gly, 76Lys, 79Arg, 85Glu, 86Ala, 92Asn, 93Val, 94Leu, 97Thr, 99Asp, 102Lys, 106Tyr, 107Ser, 110Cys, 111Ala, 114Asp, 115Lys, 117Asp, 118Ser, 120Gly, 121Ser, 128Asn, 129Asp, 130His, 133Gly, 135Ser, 138Gly, 140Leu, 145Ser, 148Asn, 151Tyr, 152Glu, 155Lys, 156His, 165Leu, 167Asp, 168Glu, 169Leu, 170Val, 171Lys, 173Asp, 184Leu, 186Gly, 187Ser, 188Asp, 190Gly, 191Thr, 195Cys, 198Ala, 200Gly, 201Gly, 204Asp, 205Ser, 209Ser, 210Cys, Ala212Thr, 215Cys, 216Arg, 217Val, 218Gly, 220Asn, 223Glu, 225Gly, 228Ile, 230Asp, 231Lys, 233Ser, 234Lys, 235Tyr, 237Tyr, 238His, 243Tyr, 244Ser, 249Thr, 255Leu, 256Ser, 260Glu, 264Arg, 267Ala, 268Glu, 272Ala, 275Val, 276Ala, 278Asp, 279Leu, 286Asp, 288Gly, 289Asp, 290Phe, 294Val, 297Gly, 300Ser, 302Val, 304Cys, 307Gln, 310Ser, 313His, 315Ile, 316Leu, 317Thr, 319Ser, 324Val, 327Leu, 331Val, Val332Ile, 335His, 337Cys, 338Phe, 340Phe, 342Gly, 343Asp, 346Ile, 351Ile, 354Asp, 360Gln, 362Leu, 363Arg, 364Glu, 365Tyr, 366Gly, 368Val, 370Thr, 371Arg, 373Asp, 374Lys, 376Glu, 377Gly, 379Leu, 380Val, 381Ile, Asn384Asp, 386Thr, 392Arg, 394Ser, 397Arg, 398Asp, 405Lys, 406Leu, 407Asp, 408Gln, 411Ile, 413Arg, 415Leu, 419Arg, 420Gly, 425Asn, 429Ser, 431Val, 433His, 439Gln, 440Leu, 443Leu, 444Leu, 446Glu, 447Ala, 451Gly, 452Pro, 457Lys, 458Val, 463Ile, 465Glu, 466Ile, 469Gly, 472Glu, 482Phe, 483Arg, 502Glu (n=205)

P6- and P7-type (141 strains)	JX989075	1Gly, 2Asn, 6Gly, 7Thr, 9Cys, 10Gly, 11Ala, 13Ile, 14Leu, 15Gly, 17Gly, 18Lys, 20Pro, 21Lys, 22Leu, 23Ser, 24Thr, 27Lys, 30Arg, 36Leu, 37Pro, 39Gly, 40Thr, 41Tyr, 42Glu, 44Ala, 47Gly, 48Gly, 51Pro, 53Val, 54Glu, 55Lys, 56Gly, 57Pro, 58Ser, 62Val, 64Arg, 65Asp, 67Leu, 68Lys, 69Pro, 70Phe, 72Glu, 74Arg, 75Gly, 76Lys, 79Arg, 80Pro, 83Leu, 85Glu, 86Ala, 87Lys, 92Asn, 93Val, 94Leu, 97Thr, 99Asp, 102Lys, 103Pro, 106Tyr, 107Ser, 110Cys, 111Ala, 112Ser, 114Asp, 115Lys, 116Thr, 117Asp, 118Ser, 120Gly, 121Ser, 123His, 128Asn, 129Asp, 130His, 132Asn, 133Gly, 135Ser, 136Phe, 137Thr, 138Gly, 139Pro, 140Leu, 142Asp, 145Ser, 148Asn, 151Tyr, 152Glu, 155Lys, 156His, 159Pro, 164Ala, 165Leu, 167Asp, 168Glu, 169Leu, 170Val, Lys171Arg, 173Asp, 175Ile, 176Tyr, Lys177Arg, 182Arg, 184Leu, 186Gly, 187Ser, 188Asp, 190Gly, 191Thr, 195Cys, 198Ala, 200Gly, 201Gly, 204Asp, 205Ser, 209Ser, 210Cys, 212Ala, 213Leu, 215Cys, 216Arg, 217Val, 218Gly, 220Asn, 223Glu, 225Gly, 226Pro, 228Ile, 230Asp, 231Lys, 233Ser, 234Lys, 235Tyr, 237Tyr, 238His, 243Tyr, 244Ser, 245Arg, 247Asp, 249Thr, 252Arg, 254Ile, 255Leu, 256Ser, 258Ala, 264Arg, 265Phe, 267Ala, 268Glu, 272Ala, 274Val, 275Val, 276Ala, 278Asp, 279Leu, 280Leu, 282Pro, 286Asp, 288Gly, 289Asp, 290Phe, 294Val, 297Gly, 300Ser, 302Val, 304Cys, 305Thr, 307Gln, 310Ser, 313His, 315Ile, 316Leu, 317Thr, 319Ser, 320Ala, 324Val, 327Leu, 328Ser, 331Val, 332Val, 335His, 337Cys, 338Phe, 339Ser, 340Phe, 342Gly, 343Asp, 346Ile, 347Val, 351Ile, 352Asn, 354Asp, 360Gln, 362Leu, 363Arg, 364Glu, 365Tyr, 366Gly, 368Val, 370Thr, 371Arg, 373Asp, 374Lys, 376Glu, 377Gly, 379Leu, 380Val, 381Ile, 383Glu, Asn384Asp, 385Leu, 386Thr, 389Thr, 391Leu, 392Arg, 393Arg, 394Ser, 397Arg, 398Asp, 404Gly, 405Lys, 406Leu, 407Asp, 408Gln, 411Ile, 413Arg, 414Gln, 415Leu, 416Tyr, 419Arg, 420Gly, 422Asn, 425Asn, 427Tyr, 429Ser, 431Val, 433His, 439Gln, 440Leu, 443Leu, 444Leu, 445Gly, 446Glu, 447Ala, 449Leu, 451Gly, 452Pro, 454Phe, 457Lys, 458Val, 463Ile, 465Glu, 466Ile, 469Gly, 472Glu, 482Phe, 483Arg, 502Glu (n=258)

	AB039777 (prototype)	1Gly, 2Asn, 6Gly, 7Thr, 9Cys, 10Gly, 11Ala, 13Ile, 14Leu, 15Gly, 17Gly, 18Lys, 20Pro, 21Lys, 22Leu, 23Ser, 24Thr, 27Lys, 30Arg, 36Leu, 37Pro, 39Gly, 40Thr, 41Tyr, 42Glu, 44Ala, 47Gly, 48Gly, 51Pro, 53Val, 54Glu, 55Lys, 56Gly, 57Pro, 58Ser, 62Val, 64Arg, 65Asp, 67Leu, 68Lys, 69Pro, 70Phe, 72Glu, 74Arg, 75Gly, 76Lys, 79Arg, 80Pro, 83Leu, 85Glu, 86Ala, 87Lys, 92Asn, 93Val, 94Leu, 97Thr, 99Asp, 102Lys, 103Pro, 106Tyr, 107Ser, 110Cys, 111Ala, 112Ser, 114Asp, 115Lys, 116Thr, 117Asp, 118Ser, 120Gly, 121Ser, 123His, 128Asn, 129Asp, 130His, 132Asn, 133Gly, 135Ser, 136Phe, 137Thr, 138Gly, 139Pro, 140Leu, 142Asp, 145Ser, 148Asn, 151Tyr, 152Glu, 155Lys, 156His, 159Pro, 164Ala, 165Leu, 167Asp, 168Glu, 169Leu, 170Val, 171Lys, 173Asp, 175Ile, 176Tyr, 177Lys, 182Arg, 184Leu, 186Gly, 187Ser, 188Asp, 190Gly, 191Thr, 195Cys, 198Ala, 200Gly, 201Gly, 204Asp, 205Ser, 209Ser, 210Cys, 212Ala, 213Leu, 215Cys, 216Arg, 217Val, 218Gly, 220Asn, 223Glu, 225Gly, 226Pro, 228Ile, 230Asp, 231Lys, 233Ser, 234Lys, 235Tyr, 237Tyr, 238His, 243Tyr, 244Ser, 245Arg, 247Asp, 249Thr, 252Arg, 254Ile, 255Leu, 256Ser, 258Ala, 264Arg, 265Phe, 267Ala, 268Glu, 272Ala, 274Val, 275Val, 276Ala, 278Asp, 279Leu, 280Leu, 282Pro, 286Asp, 288Gly, 289Asp, 290Phe, 294Val, 297Gly, 300Ser, 302Val, 304Cys, 305Thr, 307Gln, 310Ser, 313His, 315Ile, 316Leu, 317Thr, 319Ser, 320Ala, 324Val, 327Leu, 328Ser, 331Val, 332Val, 335His, 337Cys, 338Phe, 339Ser, 340Phe, 342Gly, 343Asp, 346Ile, 347Val, 351Ile, 352Asn, 354Asp, 360Gln, 362Leu, 363Arg, 364Glu, 365Tyr, 366Gly, 368Val, 370Thr, 371Arg, 373Asp, 374Lys, 376Glu, 377Gly, 379Leu, 380Val, 381Ile, 383Glu, 384Asn, 385Leu, 386Thr, 389Thr, 391Leu, 392Arg, 393Arg, 394Ser, 397Arg, 398Asp, 404Gly, 405Lys, 406Leu, 407Asp, 408Gln, 411Ile, 413Arg, 414Gln, 415Leu, 416Tyr, 419Arg, 420Gly, 422Asn, 425Asn, 427Tyr, 429Ser, 431Val, 433His, 439Gln, 440Leu, 443Leu, 444Leu, 445Gly, 446Glu, 447Ala, 449Leu, 451Gly, 452Pro, 454Phe, 457Lys, 458Val, 463Ile, 465Glu, 466Ile, 469Gly, 472Glu, 482Phe, 483Arg, 502Glu (n=258)

	LC122916	1Gly, 2Asn, 6Gly, 7Thr, 9Cys, 10Gly, 11Ala, 13Ile, 14Leu, 15Gly, 17Gly, 18Lys, 20Pro, 21Lys, 22Leu, 23Ser, 24Thr, 27Lys, 30Arg, 36Leu, 37Pro, 39Gly, 40Thr, 41Tyr, 42Glu, 44Ala, 47Gly, 48Gly, 51Pro, 53Val, 54Glu, 55Lys, 56Gly, 57Pro, 58Ser, 62Val, 64Arg, 65Asp, 67Leu, 68Lys, 69Pro, 70Phe, 72Glu, 74Arg, 75Gly, 76Lys, 79Arg, 80Pro, 83Leu, 85Glu, 86Ala, 87Lys, 92Asn, 93Val, 94Leu, 97Thr, 99Asp, 102Lys, 103Pro, 106Tyr, 107Ser, 110Cys, 111Ala, 112Ser, 114Asp, 115Lys, 116Thr, 117Asp, 118Ser, 120Gly, 121Ser, 123His, 128Asn, 129Asp, 130His, 132Asn, 133Gly, 135Ser, 136Phe, 137Thr, 138Gly, 139Pro, 140Leu, 142Asp, 145Ser, 148Asn, 151Tyr, 152Glu, 155Lys, 156His, 159Pro, 164Ala, 165Leu, 167Asp, 168Glu, 169Leu, 170Val, 171Lys, 173Asp, 175Ile, 176Tyr, 177Lys, 182Arg, 184Leu, 186Gly, 187Ser, 188Asp, 190Gly, 191Thr, 195Cys, 198Ala, 200Gly, 201Gly, 204Asp, 205Ser, 209Ser, 210Cys, 212Ala, 213Leu, 215Cys, 216Arg, 217Val, 218Gly, 220Asn, 223Glu, 225Gly, 226Pro, 228Ile, 230Asp, 231Lys, 233Ser, 234Lys, 235Tyr, 237Tyr, 238His, 243Tyr, 244Ser, 245Arg, 247Asp, 249Thr, 252Arg, 254Ile, 255Leu, 256Ser, 258Ala, 264Arg, 265Phe, 267Ala, 268Glu, 272Ala, 274Val, 275Val, 276Ala, 278Asp, 279Leu, 280Leu, 282Pro, 286Asp, 288Gly, 289Asp, 290Phe, 294Val, 297Gly, 300Ser, 302Val, 304Cys, 305Thr, 307Gln, 310Ser, 313His, 315Ile, 316Leu, 317Thr, 319Ser, 320Ala, 324Val, 327Leu, 328Ser, 331Val, 332Val, 335His, 337Cys, 338Phe, 339Ser, 340Phe, 342Gly, 343Asp, 346Ile, 347Val, 351Ile, 352Asn, 354Asp, 360Gln, 362Leu, 363Arg, 364Glu, 365Tyr, 366Gly, 368Val, 370Thr, 371Arg, 373Asp, 374Lys, 376Glu, 377Gly, 379Leu, 380Val, 381Ile, 383Glu, Asn384Asp, 385Leu, 386Thr, 389Thr, 391Leu, 392Arg, 393Arg, 394Ser, 397Arg, 398Asp, 404Gly, 405Lys, 406Leu, 407Asp, 408Gln, 411Ile, 413Arg, 414Gln, 415Leu, 416Tyr, 419Arg, 420Gly, 422Asn, 425Asn, 427Tyr, 429Ser, 431Val, 433His, 439Gln, 440Leu, 443Leu, 444Leu, 445Gly, 446Glu, 447Ala, 449Leu, 451Gly, 452Pro, 454Phe, 457Lys, 458Val, 463Ile, 465Glu, 466Ile, 469Gly, 472Glu, 482Phe, 483Arg, 502Glu (n=258)

	LC760173	1Gly, 2Asn, 6Gly, 7Thr, 9Cys, 10Gly, 11Ala, 13Ile, 14Leu, 15Gly, 17Gly, 18Lys, 20Pro, 21Lys, 22Leu, 23Ser, 24Thr, 27Lys, 30Arg, 36Leu, 37Pro, 39Gly, 40Thr, 41Tyr, 42Glu, 44Ala, 47Gly, 48Gly, 51Pro, 53Val, 54Glu, 55Lys, 56Gly, 57Pro, 58Ser, 62Val, 64Arg, 65Asp, 67Leu, 68Lys, 69Pro, 70Phe, 72Glu, 74Arg, 75Gly, 76Lys, 79Arg, 80Pro, 83Leu, 85Glu, 86Ala, 87Lys, 92Asn, 93Val, 94Leu, 97Thr, 99Asp, 102Lys, 103Pro, 106Tyr, 107Ser, 110Cys, 111Ala, 112Ser, 114Asp, 115Lys, 116Thr, 117Asp, 118Ser, 120Gly, 121Ser, 123His, 128Asn, 129Asp, 130His, 132Asn, 133Gly, 135Ser, 136Phe, 137Thr, 138Gly, 139Pro, 140Leu, 142Asp, 145Ser, 148Asn, 151Tyr, 152Glu, 155Lys, 156His, 159Pro, 164Ala, 165Leu, 167Asp, 168Glu, 169Leu, 170Val, 171Lys, 173Asp, 175Ile, 176Tyr, 177Lys, 182Arg, 184Leu, 186Gly, 187Ser, 188Asp, 190Gly, 191Thr, 195Cys, 198Ala, 200Gly, 201Gly, 204Asp, 205Ser, 209Ser, 210Cys, 212Ala, 213Leu, 215Cys, 216Arg, 217Val, 218Gly, 220Asn, 223Glu, 225Gly, 226Pro, 228Ile, 230Asp, 231Lys, 233Ser, 234Lys, 235Tyr, 237Tyr, 238His, 243Tyr, 244Ser, 245Arg, 247Asp, 249Thr, 252Arg, 254Ile, 255Leu, 256Ser, 258Ala, 264Arg, 265Phe, 267Ala, 268Glu, 272Ala, 274Val, 275Val, 276Ala, 278Asp, 279Leu, 280Leu, 282Pro, 286Asp, 288Gly, 289Asp, 290Phe, 294Val, 297Gly, 300Ser, 302Val, 304Cys, 305Thr, 307Gln, 310Ser, 313His, 315Ile, 316Leu, 317Thr, 319Ser, 320Ala, 324Val, 327Leu, 328Ser, 331Val, 332Val, 335His, 337Cys, 338Phe, 339Ser, 340Phe, 342Gly, 343Asp, 346Ile, 347Val, 351Ile, 352Asn, 354Asp, 360Gln, 362Leu, 363Arg, 364Glu, 365Tyr, 366Gly, Val368Ala, 370Thr, 371Arg, 373Asp, 374Lys, 376Glu, 377Gly, 379Leu, 380Val, 381Ile, 383Glu, Asn384Asp, 385Leu, 386Thr, 389Thr, 391Leu, 392Arg, 393Arg, 394Ser, 397Arg, 398Asp, 404Gly, 405Lys, 406Leu, 407Asp, 408Gln, 411Ile, 413Arg, 414Gln, 415Leu, 416Tyr, 419Arg, 420Gly, 422Asn, 425Asn, 427Tyr, 429Ser, 431Val, 433His, 439Gln, 440Leu, 443Leu, 444Leu, 445Gly, 446Glu, 447Ala, 449Leu, 451Gly, 452Pro, 454Phe, 457Lys, 458Val, 463Ile, 465Glu, 466Ile, 469Gly, 472Glu, 482Phe, 483Arg, 502Glu (n=258)

	MK956199	1Gly, 2Asn, 6Gly, 7Thr, 9Cys, 10Gly, 11Ala, 13Ile, 14Leu, 15Gly, 17Gly, 18Lys, 20Pro, 21Lys, 22Leu, 23Ser, 24Thr, 27Lys, 30Arg, 36Leu, 37Pro, 39Gly, 40Thr, 41Tyr, 42Glu, 44Ala, 47Gly, 48Gly, 51Pro, 53Val, 54Glu, 55Lys, 56Gly, 57Pro, 58Ser, 62Val, 64Arg, 65Asp, 67Leu, 68Lys, 69Pro, 70Phe, 72Glu, 74Arg, 75Gly, 76Lys, 79Arg, 80Pro, 83Leu, 85Glu, 86Ala, 87Lys, 92Asn, 93Val, 94Leu, 97Thr, 99Asp, 102Lys, 103Pro, 106Tyr, 107Ser, 110Cys, 111Ala, 112Ser, 114Asp, 115Lys, 116Thr, 117Asp, 118Ser, 120Gly, 121Ser, 123His, 128Asn, 129Asp, 130His, 132Asn, 133Gly, 135Ser, 136Phe, 137Thr, 138Gly, 139Pro, 140Leu, 142Asp, 145Ser, 148Asn, 151Tyr, 152Glu, 155Lys, 156His, 159Pro, 164Ala, 165Leu, 167Asp, 168Glu, 169Leu, 170Val, 171Lys, 173Asp, 175Ile, 176Tyr, Lys177Arg, 182Arg, 184Leu, 186Gly, 187Ser, 188Asp, 190Gly, 191Thr, 195Cys, 198Ala, 200Gly, 201Gly, 204Asp, 205Ser, 209Ser, 210Cys, Ala212Thr, 213Leu, 215Cys, 216Arg, 217Val, 218Gly, 220Asn, 223Glu, 225Gly, 226Pro, 228Ile, 230Asp, 231Lys, 233Ser, 234Lys, 235Tyr, 237Tyr, 238His, 243Tyr, 244Ser, 245Arg, 247Asp, 249Thr, 252Arg, 254Ile, 255Leu, 256Ser, 258Ala, 264Arg, 265Phe, 267Ala, 268Glu, 272Ala, 274Val, 275Val, 276Ala, 278Asp, 279Leu, 280Leu, 282Pro, 286Asp, 288Gly, 289Asp, 290Phe, 294Val, 297Gly, 300Ser, 302Val, 304Cys, 305Thr, 307Gln, 310Ser, 313His, 315Ile, 316Leu, 317Thr, 319Ser, 320Ala, 324Val, 327Leu, 328Ser, 331Val, Val332Ile, 335His, 337Cys, 338Phe, 339Ser, 340Phe, 342Gly, 343Asp, 346Ile, 347Val, 351Ile, 352Asn, 354Asp, 360Gln, 362Leu, 363Arg, 364Glu, 365Tyr, 366Gly, 368Val, 370Thr, 371Arg, 373Asp, 374Lys, 376Glu, 377Gly, 379Leu, 380Val, 381Ile, 383Glu, Asn384Asp, 385Leu, 386Thr, 389Thr, 391Leu, 392Arg, 393Arg, 394Ser, 397Arg, 398Asp, 404Gly, 405Lys, 406Leu, 407Asp, 408Gln, 411Ile, 413Arg, 414Gln, 415Leu, 416Tyr, 419Arg, 420Gly, 422Asn, 425Asn, 427Tyr, 429Ser, 431Val, 433His, 439Gln, 440Leu, 443Leu, 444Leu, 445Gly, 446Glu, 447Ala, 449Leu, 451Gly, 452Pro, 454Phe, 457Lys, 458Val, 463Ile, 465Glu, 466Ile, 469Gly, 472Glu, 482Phe, 483Arg, 502Glu (n=258)

Table S3b. Details of GII.P6-GII.6 and GII.P7-GII.6 *VP1* gene negative selection sites in comparing with each genotype.

VP1 genotype	Representative strain	The number of amino acid residues
P6-type *VP1* gene (15 strains)	JX989075	154Val, 226Phe, 266Thr, 362Thr, Ala367Val, Val403Ile, 525Phe, 534Phe (n=8)
P7-type *VP1* gene (126 strains)	AB039777 (prototype)	8Ala, 12Asn, 32Val, 37Ile, 38Ala, 41Val, 43Gly, 46Asn, 57Val, 62Gly, 64Phe, 65Thr, 66Val, 69Arg, 70Asn, 72Pro, 74Glu, 76Leu, 77Leu, 78Asn, 80Glu, 81Leu, 84Glu, 85Leu, 87Pro, 88Tyr, 89Leu, 91His, 94Pro, 96Tyr, 97Asn, 98Gly, 102Gly, 112Asn, 117Gly, 124Val, 128Phe, 129Pro, 130Val, 131Asp, 132Asn, 133Ile, 136Ala, 138Ile, 139Thr, 144Val, 148Val, 149arg, 152Glu, 154Val, 156Leu, 158Leu, 159Pro, 160Asp, 161Ile, 163Asn, 164Arg, 166Phe, 168Tyr, 169Asn, 171Glu, 172Asn, 173Thr, 175Arg, 177Arg, 178Leu, 180Ala, 182Leu, 183Tyr, 184Thr, 185Pro, 186Leu, 187Arg, 188Ala, 189Asn, 191Gly, 193Asp, 195Phe, 196Thr, 197Val, 199Cys, 200Arg, 201Val, 202Leu, 203Thr, 204Arg, 205Pro, 207Pro, 208Asp, 210Glu, 211Phe, 212Thr, 213Phe, 214Leu, 215Val, 218Thr, 219Val, 220Glu, 223Thr, 224Lys, 225Pro, 226Phe, 228Leu, 229Pro, 230Ile, 231Leu, 234Gly, 235Glu, 236Leu, 237Ser, 238Asn, 239Ser, 240Arg, 242Pro, 244Ala, 246Asp, 248Leu, 249Tyr, 250Thr, 251Asp, 252Pro, 254Glu, 257Val, 258Val, 262Asn, 263Gly, 264Arg, 265Cys, 267Leu, 268Asp, 270Thr, 271Leu, 272Gln, 273Gly, 276Gln, 278Val, 281Gln, 285Phe, 286Arg, 287Gly, 289Leu, 292Gln, 295Arg, 298Asp, 301Asp, 305Arg, 309His, 310Pro, 311Leu, 312His, 313Val, 320Gly, 323Tyr, 324Asp, 325Pro, 326Thr, 328Asp, 330Pro, 331Ala, 333Leu, 334Gly, 337Asp, 339Lys, 340Gly, 341Thr, 342Val, 344Gly, 347Ser, 348Gln, 349Arg, 350Asp, 353Gly, 359His, 361Ala, 364Ala, 366Glu, 369Ile, 371Thr, 373Asp, 376Tyr, 379Lys, 380Leu, 381Gly, 383Ile, 384Leu, 389Ser, 392Phe, 396Gln, 397Pro, 399Arg, 400Phe, 401Thr, 402Pro, 404Gly, 406Gly, 408Asn, 412Gln, 415Leu, 416Pro, 417Asp, 418Tyr, 420Gly, 422Leu, 423Thr, 424Leu, 425Asn, 428Leu, 430Pro, 431Ala, 432Val, 433Ser, 435Ser, 436Phe, 437Pro, 440Arg, 441Ile, 442Leu, 444Phe, 445Arg, 446Ser, 448Val, 449Pro, 452Gly, 454Tyr, 455Gly, 457Gly, 460Asp, 466Glu, 470His, 472Tyr, 473Gln, 474Glu, 475Ala, 476Ala, 479Gln, 480Ser, 481Ala, 482Val, 483Ala, 484Leu, 485Val, 486Arg, 488Val, 489Asn, 490Pro, 491Asp, 494Arg, 499Ala, 500Lys, 501Leu, 502His, 504Glu, 505Gly, 510Ala, 513Gly, 514Asn, 515Asn, 516Pro, 518Val, 519Val, 523Gly, 524Tyr,

		525Phe, 526Arg, 527Phe, 531Val, 532Asn, 533Gln, 534Phe, 535Tyr, 537Leu, 543Gly, 548Arg, 550Gln (n=274)
	LC122916	8Ala, 12Asn, 32Val, 37Ile, 38Ala, 41Val, 43Gly, 46Asn, 57Val, 62Gly, 64Phe, 65Thr, 66Val, 69Arg, 70Asn, 72Pro, 74Glu, 76Leu, 77Leu, 78Asn, 80Glu, 81Leu, 84Glu, 85Leu, 87Pro, 88Tyr, 89Leu, 91His, Pro94Arg, 96Tyr, 97Asn, 98Gly, 102Gly, 112Asn, 117Gly, 124Val, 128Phe, 129Pro, 130Val, 131Asp, 132Asn, 133Ile, 136Ala, 138Ile, 139Thr, 144Val, 148Val, 149arg, 152Glu, 154Val, 156Leu, 158Leu, 159Pro, 160Asp, 161Ile, 163Asn, 164Arg, 166Phe, 168Tyr, 169Asn, 171Glu, Asn172Asp, 173Thr, 175Arg, 177Arg, 178Leu, 180Ala, 182Leu, 183Tyr, 184Thr, 185Pro, 186Leu, 187Arg, 188Ala, 189Asn, 191Gly, 193Asp, 195Phe, 196Thr, 197Val, 199Cys, 200Arg, 201Val, 202Leu, 203Thr, 204Arg, 205Pro, 207Pro, 208Asp, 210Glu, 211Phe, 212Thr, 213Phe, 214Leu, 215Val, 218Thr, 219Val, 220Glu, 223Thr, 224Lys, 225Pro, 226Phe, 228Leu, 229Pro, 230Ile, 231Leu, 234Gly, 235Glu, 236Leu, 237Ser, 238Asn, 239Ser, 240Arg, 242Pro, 244Ala, 246Asp, 248Leu, 249Tyr, 250Thr, 251Asp, 252Pro, 254Glu, 257Val, 258Val, 262Asn, 263Gly, 264Arg, 265Cys, 267Leu, 268Asp, 270Thr, 271Leu, 272Gln, 273Gly, 276Gln, 278Val, 281Gln, 285Phe, 286Arg, 287Gly, 289Leu, 292Gln, 295Arg, 298Asp, 301Asp, 305Arg, 309His, 310Pro, 311Leu, 312His, 313Val, 320Gly, 323Tyr, 324Asp, 325Pro, 326Thr, 328Asp, 330Pro, 331Ala, 333Leu, 334Gly, 337Asp, 339Lys, 340Gly, 341Thr, 342Val, 344Gly, 347Ser, 348Gln, 349Arg, 350Asp, 353Gly, 359His, 361Ala, 364Ala, 366Glu, 369Ile, 371Thr, 373Asp, 376Tyr, 379Lys, 380Leu, 381Gly, 383Ile, 384Leu, 389Ser, 392Phe, 396Gln, 397Pro, 399Arg, 400Phe, 401Thr, 402Pro, 404Gly, 406Gly, 408Asn, 412Gln, 415Leu, 416Pro, 417Asp, 418Tyr, 420Gly, 422Leu, 423Thr, 424Leu, 425Asn, 428Leu, 430Pro, 431Ala, 432Val, 433Ser, 435Ser, 436Phe, 437Pro, 440Arg, 441Ile, 442Leu, 444Phe, 445Arg, 446Ser, 448Val, 449Pro, 452Gly, 454Tyr, 455Gly, 457Gly, 460Asp, 466Glu, 470His, 472Tyr, 473Gln, 474Glu, 475Ala, 476Ala, 479Gln, 480Ser, 481Ala, 482Val, 483Ala, 484Leu, 485Val, 486Arg, 488Val, 489Asn, 490Pro, 491Asp, 494Arg, 499Ala, 500Lys, 501Leu, 502His, 504Glu, 505Gly, 510Ala, 513Gly, 514Asn, 515Asn, 516Pro, 518Val, 519Val, 523Gly, 524Tyr, 525Phe, 526Arg, 527Phe, 531Val, 532Asn, 533Gln, 534Phe, 535Tyr, 537Leu, 543Gly, 548Arg,

		550Gln (n=274)
	MH791993	8Ala, 12Asn, 32Val, 37Ile, 38Ala, 41Val, 43Gly, 46Asn, 57Val, 62Gly, 64Phe, 65Thr, 66Val, 69Arg, 70Asn, 72Pro, 74Glu, 76Leu, 77Leu, 78Asn, 80Glu, 81Leu, 84Glu, 85Leu, 87Pro, 88Tyr, 89Leu, 91His, Pro94Arg, 96Tyr, 97Asn, 98Gly, 102Gly, 112Asn, 117Gly, 124Val, 128Phe, 129Pro, 130Val, Asp131Glu, 132Asn, 133Ile, 136Ala, 138Ile, 139Thr, 144Val, 148Val, 149arg, 152Glu, 154Val, 156Leu, 158Leu, 159Pro, 160Asp, 161Ile, 163Asn, 164Arg, 166Phe, 168Tyr, 169Asn, 171Glu, 172Asn, 173Thr, 175Arg, 177Arg, 178Leu, 180Ala, 182Leu, 183Tyr, 184Thr, 185Pro, 186Leu, 187Arg, 188Ala, 189Asn, 191Gly, 193Asp, 195Phe, 196Thr, 197Val, 199Cys, 200Arg, 201Val, 202Leu, 203Thr, 204Arg, 205Pro, 207Pro, 208Asp, 210Glu, 211Phe, 212Thr, 213Phe, 214Leu, 215Val, 218Thr, 219Val, 220Glu, 223Thr, 224Lys, 225Pro, 226Phe, 228Leu, 229Pro, 230Ile, 231Leu, 234Gly, 235Glu, 236Leu, 237Ser, 238Asn, 239Ser, 240Arg, 242Pro, Ala244Pro, 246Asp, 248Leu, 249Tyr, 250Thr, 251Asp, 252Pro, 254Glu, 257Val, 258Val, 262Asn, 263Gly, 264Arg, 265Cys, 267Leu, 268Asp, 270Thr, 271Leu, 272Gln, 273Gly, 276Gln, 278Val, 281Gln, 285Phe, 286Arg, 287Gly, 289Leu, 292Gln, 295Arg, 298Asp, 301Asp, 305Arg, 309His, 310Pro, 311Leu, 312His, 313Val, 320Gly, 323Tyr, 324Asp, 325Pro, 326Thr, Asp328Glu, 330Pro, 331Ala, 333Leu, 334Gly, 337Asp, 339Lys, 340Gly, 341Thr, 342Val, 344Gly, 347Ser, 348Gln, 349Arg, Asp350Asn, 353Gly, 359His, 361Ala, 364Ala, 366Glu, 369Ile, 371Thr, Asp373Asn, 376Tyr, 379Lys, 380Leu, 381Gly, Ile383Val, 384Leu, 389Ser, 392Phe, 396Gln, 397Pro, 399Arg, 400Phe, 401Thr, 402Pro, 404Gly, 406Gly, Asn408Asp, 412Gln, 415Leu, 416Pro, Asp417Glu, 418Tyr, 420Gly, 422Leu, 423Thr, 424Leu, 425Asn, 428Leu, 430Pro, 431Ala, 432Val, Ser433Ala, Ser435Ala, 436Phe, 437Pro, 440Arg, 441Ile, 442Leu, 444Phe, 445Arg, 446Ser, 448Val, 449Pro, 452Gly, 454Tyr, 455Gly, 457Gly, 460Asp, 466Glu, 470His, 472Tyr, 473Gln, 474Glu, 475Ala, 476Ala, 479Gln, 480Ser, 481Ala, 482Val, 483Ala, 484Leu, Val485Ile, 486Arg, 488Val, 489Asn, 490Pro, 491Asp, 494Arg, 499Ala, 500Lys, 501Leu, 502His, 504Glu, 505Gly, 510Ala, 513Gly, 514Asn, 515Asn, 516Pro, 518Val, 519Val, 523Gly, 524Tyr, 525Phe, 526Arg, 527Phe, 531Val, 532Asn, 533Gln, 534Phe, 535Tyr, 537Leu, 543Gly, 548Arg, 550Gln (n=274)

	MK956199	8Ala, 12Asn, 32Val, 37Ile, 38Ala, 41Val, 43Gly, 46Asn, 57Val, 62Gly, 64Phe, 65Thr, 66Val, 69Arg, 70Asn, 72Pro, 74Glu, 76Leu, 77Leu, 78Asn, 80Glu, 81Leu, 84Glu, 85Leu, 87Pro, 88Tyr, 89Leu, 91His, 94Pro, 96Tyr, 97Asn, 98Gly, 102Gly, 112Asn, 117Gly, 124Val, 128Phe, 129Pro, 130Val, Asp131Glu, 132Asn, 133Ile, 136Ala, 138Ile, 139Thr, 144Val, 148Val, 149arg, 152Glu, 154Val, 156Leu, 158Leu, 159Pro, 160Asp, 161Ile, 163Asn, 164Arg, 166Phe, 168Tyr, 169Asn, 171Glu, 172Asn, 173Thr, 175Arg, 177Arg, 178Leu, 180Ala, 182Leu, 183Tyr, 184Thr, 185Pro, 186Leu, 187Arg, 188Ala, 189Asn, 191Gly, 193Asp, 195Phe, 196Thr, 197Val, 199Cys, 200Arg, 201Val, 202Leu, 203Thr, 204Arg, 205Pro, 207Pro, 208Asp, 210Glu, 211Phe, 212Thr, 213Phe, 214Leu, 215Val, 218Thr, 219Val, 220Glu, 223Thr, 224Lys, 225Pro, 226Phe, 228Leu, 229Pro, 230Ile, 231Leu, 234Gly, 235Glu, 236Leu, 237Ser, 238Asn, 239Ser, 240Arg, 242Pro, Ala244Pro, 246Asp, 248Leu, 249Tyr, 250Thr, 251Asp, 252Pro, 254Glu, 257Val, 258Val, 262Asn, 263Gly, 264Arg, 265Cys, 267Leu, 268Asp, 270Thr, 271Leu, 272Gln, 273Gly, 276Gln, 278Val, 281Gln, 285Phe, 286Arg, 287Gly, 289Leu, 292Gln, 295Arg, 298Asp, 301Asp, 305Arg, 309His, 310Pro, 311Leu, 312His, 313Val, 320Gly, 323Tyr, 324Asp, 325Pro, 326Thr, Asp328Glu, 330Pro, 331Ala, 333Leu, 334Gly, 337Asp, 339Lys, 340Gly, 341Thr, 342Val, 344Gly, 347Ser, 348Gln, 349Arg, 350Asp, 353Gly, 359His, 361Ala, 364Ala, 366Glu, 369Ile, 371Thr, 373Asp, 376Tyr, 379Lys, 380Leu, 381Gly, 383Ile, 384Leu, 389Ser, 392Phe, 396Gln, 397Pro, 399Arg, 400Phe, 401Thr, 402Pro, 404Gly, 406Gly, 408Asn, 412Gln, 415Leu, 416Pro, 417Asp, 418Tyr, 420Gly, 422Leu, 423Thr, 424Leu, 425Asn, 428Leu, 430Pro, 431Ala, 432Val, Ser433Ala, Ser435Ala, 436Phe, 437Pro, 440Arg, 441Ile, 442Leu, 444Phe, 445Arg, 446Ser, 448Val, 449Pro, 452Gly, 454Tyr, 455Gly, 457Gly, 460Asp, 466Glu, 470His, 472Tyr, 473Gln, 474Glu, 475Ala, 476Ala, 479Gln, 480Ser, 481Ala, 482Val, 483Ala, 484Leu, Val485Ile, 486Arg, 488Val, 489Asn, 490Pro, 491Asp, 494Arg, 499Ala, 500Lys, 501Leu, 502His, 504Glu, 505Gly, 510Ala, 513Gly, 514Asn, 515Asn, 516Pro, 518Val, 519Val, 523Gly, 524Tyr, 525Phe, 526Arg, 527Phe, 531Val, 532Asn, 533Gln, 534Phe, 535Tyr, 537Leu, 543Gly, 548Arg, 550Gln (n=274)

P6- and P7-type *VP1* genes (141 strains)	JX989075	8Ala, 11Ser, 12Asn, 32Val, 37Ile, 38Ala, 41Val, 43Gly, 45Gln, 46Asn, 57Val, 62Gly, 64Phe, 65Thr, 66Val, 69Arg, 70Asn, 72Pro, 74Glu, 76Leu, 77Leu, 78Asn, 80Glu, 81Leu, 84Glu, 85Leu, 86Asn, 87Pro, 88Tyr, 89Leu, 91His, 92Leu, Pro94Arg, 96Tyr, 97Asn, 98Gly, 102Gly, 112Asn, 117Gly, 124Val, 128Phe, 129Pro, 130Val, Asp131Glu, 132Asn, 133Ile, 136Ala, 138Ile, 139Thr, 144Val, 148Val, 149arg, 150Gln, 152Glu, 154Val, 156Leu, 158Leu, 159Pro, 160Asp, 161Ile, 163Asn, 164Arg, 166Phe, 168Tyr, 169Asn, 171Glu, Asn172Asp, 173Thr, 175Arg, 177Arg, 178Leu, 179Val, 180Ala, 182Leu, 183Tyr, 184Thr, 185Pro, 186Leu, 187Arg, 188Ala, 189Asn, 191Gly, 193Asp, 195Phe, 196Thr, 197Val, 199Cys, 200Arg, 201Val, 202Leu, 203Thr, 204Arg, 205Pro, 207Pro, 208Asp, 210Glu, 211Phe, 212Thr, 213Phe, 214Leu, 215Val, 218Thr, 219Val, 220Glu, 223Thr, 224Lys, 225Pro, 226Phe, 228Leu, 229Pro, 230Ile, 231Leu, 232Thr, 234Gly, 235Glu, 236Leu, 237Ser, 238Asn, 239Ser, 240Arg, 241Phe, 242Pro, Ala244Pro, 246Asp, 248Leu, 249Tyr, 250Thr, 251Asp, 252Pro, 254Glu, 256Ile, 257Val, 258Val, 262Asn, 263Gly, 264Arg, 265Cys, 266Thr, 267Leu, 268Asp, 269Gly, 270Thr, 271Leu, 272Gln, 273Gly, 276Gln, 277Leu, 278Val, 281Gln, 285Phe, 286Arg, 287Gly, 289Leu, 292Gln, 295Arg, 298Asp, 301Asp, Pro303Ala, 305Arg, 309His, 310Pro, 311Leu, 312His, 313Val, 318Leu, 320Gly, 323Tyr, 324Asp, 325Pro, 326Thr, Asp328Glu, Ile329Val, 330Pro, 331Ala, 333Leu, 334Gly, 337Asp, 338Phe, 339Lys, 340Gly, 341Thr, 342Val, 344Gly, 347Ser, 348Gln, 349Arg, 353Gly, 359His, 361Ala, 362Thr, 363Arg, 364Ala, 366Glu, 369Ile, 370Asp, 371Thr, 376Tyr, 379Lys, 380Leu, 381Gly, 383Ile, 384Leu, 389Ser, 391Asp, 392Phe, 396Gln, 397Pro, 400Phe, 401Thr, 402Pro, Val403Ile, 404Gly, 406Gly, 412Gln, 415Leu, 416Pro, Asp417Glu, 418Tyr, 420Gly, 422Leu, 423Thr, 424Leu, 425Asn, 428Leu, 430Pro, 431Ala, 432Val, Ser433Ala, Ser435Ala, 436Phe, 437Pro, 440Arg, 441Ile, 442Leu, 444Phe, 445Arg, 446Ser, 448Val, 449Pro, 452Gly, 453Gly, 454Tyr, 455Gly, 456Ser, 457Gly, 460Asp, 466Glu, 470His, 472Tyr, 473Gln, 474Glu, 475Ala, 476Ala, 477Pro, 479Gln, 480Ser, 481Ala, 482Val, 483Ala, 484Leu, Val485Ile, 486Arg, 488Val, 489Asn, 490Pro, 491Asp, 494Arg, 499Ala, 500Lys, 501Leu, 502His, 504Glu, 505Gly, 510Ala, 513Gly, 514Asn, 515Asn, 516Pro, 518Val, 519Val, 521Pro, 523Gly, 524Tyr, 525Phe, 526Arg, 527Phe, 529Ala, 531Val, 532Asn, 533Gln, 534Phe, 535Tyr, 537Leu, 543Gly, 546Arg, 547Arg, 548Arg, 550Gln (n=298)

	AB039777 (prototype)	8Ala, 11Ser, 12Asn, 32Val, 37Ile, 38Ala, 41Val, 43Gly, 45Gln, 46Asn, 57Val, 62Gly, 64Phe, 65Thr, 66Val, 69Arg, 70Asn, 72Pro, 74Glu, 76Leu, 77Leu, 78Asn, 80Glu, 81Leu, 84Glu, 85Leu, 86Asn, 87Pro, 88Tyr, 89Leu, 91His, 92Leu, 94Pro, 96Tyr, 97Asn, 98Gly, 102Gly, 112Asn, 117Gly, 124Val, 128Phe, 129Pro, 130Val, 131Asp, 132Asn, 133Ile, 136Ala, 138Ile, 139Thr, 144Val, 148Val, 149arg, 150Gln, 152Glu, 154Val, 156Leu, 158Leu, 159Pro, 160Asp, 161Ile, 163Asn, 164Arg, 166Phe, 168Tyr, 169Asn, 171Glu, 172Asn, 173Thr, 175Arg, 177Arg, 178Leu, 179Val, 180Ala, 182Leu, 183Tyr, 184Thr, 185Pro, 186Leu, 187Arg, 188Ala, 189Asn, 191Gly, 193Asp, 195Phe, 196Thr, 197Val, 199Cys, 200Arg, 201Val, 202Leu, 203Thr, 204Arg, 205Pro, 207Pro, 208Asp, 210Glu, 211Phe, 212Thr, 213Phe, 214Leu, 215Val, 218Thr, 219Val, 220Glu, 223Thr, 224Lys, 225Pro, 226Phe, 228Leu, 229Pro, 230Ile, 231Leu, 232Thr, 234Gly, 235Glu, 236Leu, 237Ser, 238Asn, 239Ser, 240Arg, 241Phe, 242Pro, 244Ala, 246Asp, 248Leu, 249Tyr, 250Thr, 251Asp, 252Pro, 254Glu, 256Ile, 257Val, 258Val, 262Asn, 263Gly, 264Arg, 265Cys, 266Thr, 267Leu, 268Asp, 269Gly, 270Thr, 271Leu, 272Gln, 273Gly, 276Gln, 277Leu, 278Val, 281Gln, 285Phe, 286Arg, 287Gly, 289Leu, 292Gln, 295Arg, 298Asp, 301Asp, 303Pro, 305Arg, 309His, 310Pro, 311Leu, 312His, 313Val, 318Leu, 320Gly, 323Tyr, 324Asp, 325Pro, 326Thr, 328Asp, 329Ile, 330Pro, 331Ala, 333Leu, 334Gly, 337Asp, 338Phe, 339Lys, 340Gly, 341Thr, 342Val, 344Gly, 347Ser, 348Gln, 349Arg, 353Gly, 359His, 361Ala, 362Thr, 363Arg, 364Ala, 366Glu, 369Ile, 370Asp, 371Thr, 376Tyr, 379Lys, 380Leu, 381Gly, 383Ile, 384Leu, 389Ser, 391Asp, 392Phe, 396Gln, 397Pro, 400Phe, 401Thr, 402Pro, 403Val, 404Gly, 406Gly, 412Gln, 415Leu, 416Pro, 417Asp, 418Tyr, 420Gly, 422Leu, 423Thr, 424Leu, 425Asn, 428Leu, 430Pro, 431Ala, 432Val, 433Ser, 435Ser, 436Phe, 437Pro, 440Arg, 441Ile, 442Leu, 444Phe, 445Arg, 446Ser, 448Val, 449Pro, 452Gly, 453Gly, 454Tyr, 455Gly, 456Ser, 457Gly, 460Asp, 466Glu, 470His, 472Tyr, 473Gln, 474Glu, 475Ala, 476Ala, 477Pro, 479Gln, 480Ser, 481Ala, 482Val, 483Ala, 484Leu, 485Val, 486Arg, 488Val, 489Asn, 490Pro, 491Asp, 494Arg, 499Ala, 500Lys, 501Leu, 502His, 504Glu, 505Gly, 510Ala, 513Gly, 514Asn, 515Asn, 516Pro, 518Val, 519Val, 521Pro, 523Gly, 524Tyr, 525Phe, 526Arg, 527Phe, 529Ala, 531Val, 532Asn, 533Gln, 534Phe, 535Tyr, 537Leu, 543Gly, 546Arg, 547Arg, 548Arg, 550Gln (n=298)

	LC122916	8Ala, 11Ser, 12Asn, 32Val, 37Ile, 38Ala, 41Val, 43Gly, 45Gln, 46Asn, 57Val, 62Gly, 64Phe, 65Thr, 66Val, 69Arg, 70Asn, 72Pro, 74Glu, 76Leu, 77Leu, 78Asn, 80Glu, 81Leu, 84Glu, 85Leu, 86Asn, 87Pro, 88Tyr, 89Leu, 91His, 92Leu, Pro94Arg, 96Tyr, 97Asn, 98Gly, 102Gly, 112Asn, 117Gly, 124Val, 128Phe, 129Pro, 130Val, Asp131Glu, 132Asn, 133Ile, 136Ala, 138Ile, 139Thr, 144Val, 148Val, 149arg, 150Gln, 152Glu, 154Val, 156Leu, 158Leu, 159Pro, 160Asp, 161Ile, 163Asn, 164Arg, 166Phe, 168Tyr, 169Asn, 171Glu, Asn172Asp, 173Thr, 175Arg, 177Arg, 178Leu, 179Val, 180Ala, 182Leu, 183Tyr, 184Thr, 185Pro, 186Leu, 187Arg, 188Ala, 189Asn, 191Gly, 193Asp, 195Phe, 196Thr, 197Val, 199Cys, 200Arg, 201Val, 202Leu, 203Thr, 204Arg, 205Pro, 207Pro, 208Asp, 210Glu, 211Phe, 212Thr, 213Phe, 214Leu, 215Val, 218Thr, 219Val, 220Glu, 223Thr, 224Lys, 225Pro, 226Phe, 228Leu, 229Pro, 230Ile, 231Leu, 232Thr, 234Gly, 235Glu, 236Leu, 237Ser, 238Asn, 239Ser, 240Arg, 241Phe, 242Pro, 244Ala, 246Asp, 248Leu, 249Tyr, 250Thr, 251Asp, 252Pro, 254Glu, 256Ile, 257Val, 258Val, 262Asn, 263Gly, 264Arg, 265Cys, 266Thr, 267Leu, 268Asp, 269Gly, 270Thr, 271Leu, 272Gln, 273Gly, 276Gln, 277Leu, 278Val, 281Gln, 285Phe, 286Arg, 287Gly, 289Leu, 292Gln, 295Arg, 298Asp, 301Asp, 303Pro, 305Arg, 309His, 310Pro, 311Leu, 312His, 313Val, 318Leu, 320Gly, 323Tyr, 324Asp, 325Pro, 326Thr, 328Asp, 329Ile, 330Pro, 331Ala, 333Leu, 334Gly, 337Asp, 338Phe, 339Lys, 340Gly, 341Thr, 342Val, 344Gly, 347Ser, 348Gln, 349Arg, 353Gly, 359His, 361Ala, 362Thr, 363Arg, 364Ala, 366Glu, 369Ile, 370Asp, 371Thr, 376Tyr, 379Lys, 380Leu, 381Gly, 383Ile, 384Leu, 389Ser, 391Asp, 392Phe, 396Gln, 397Pro, 400Phe, 401Thr, 402Pro, 403Val, 404Gly, 406Gly, 412Gln, 415Leu, 416Pro, 417Asp, 418Tyr, 420Gly, 422Leu, 423Thr, 424Leu, 425Asn, 428Leu, 430Pro, 431Ala, 432Val, 433Ser, 435Ser, 436Phe, 437Pro, 440Arg, 441Ile, 442Leu, 444Phe, 445Arg, 446Ser, 448Val, 449Pro, 452Gly, 453Gly, 454Tyr, 455Gly, 456Ser, 457Gly, 460Asp, 466Glu, 470His, 472Tyr, 473Gln, 474Glu, 475Ala, 476Ala, 477Pro, 479Gln, 480Ser, 481Ala, 482Val, 483Ala, 484Leu, 485Val, 486Arg, 488Val, 489Asn, 490Pro, 491Asp, 494Arg, 499Ala, 500Lys, 501Leu, 502His, 504Glu, 505Gly, 510Ala, 513Gly, 514Asn, 515Asn, 516Pro, 518Val, 519Val, 521Pro, 523Gly, 524Tyr, 525Phe, 526Arg, 527Phe, 529Ala, 531Val, 532Asn, 533Gln, 534Phe, 535Tyr, 537Leu, 543Gly, 546Arg, 547Arg, 548Arg, 550Gln (n=298)

	MK791993	8Ala, 11Ser, 12Asn, 32Val, 37Ile, 38Ala, 41Val, 43Gly, 45Gln, 46Asn, 57Val, 62Gly, 64Phe, 65Thr, 66Val, 69Arg, 70Asn, 72Pro, 74Glu, 76Leu, 77Leu, 78Asn, 80Glu, 81Leu, 84Glu, 85Leu, 86Asn, 87Pro, 88Tyr, 89Leu, 91His, 92Leu, Pro94Arg, 96Tyr, 97Asn, 98Gly, 102Gly, 112Asn, 117Gly, 124Val, 128Phe, 129Pro, 130Val, Asp131Glu, 132Asn, 133Ile, 136Ala, 138Ile, 139Thr, 144Val, 148Val, 149arg, 150Gln, 152Glu, 154Val, 156Leu, 158Leu, 159Pro, 160Asp, 161Ile, 163Asn, 164Arg, 166Phe, 168Tyr, 169Asn, 171Glu, 172Asn, 173Thr, 175Arg, 177Arg, 178Leu, 179Val, 180Ala, 182Leu, 183Tyr, 184Thr, 185Pro, 186Leu, 187Arg, 188Ala, 189Asn, 191Gly, 193Asp, 195Phe, 196Thr, 197Val, 199Cys, 200Arg, 201Val, 202Leu, 203Thr, 204Arg, 205Pro, 207Pro, 208Asp, 210Glu, 211Phe, 212Thr, 213Phe, 214Leu, 215Val, 218Thr, 219Val, 220Glu, 223Thr, 224Lys, 225Pro, 226Phe, 228Leu, 229Pro, 230Ile, 231Leu, 232Thr, 234Gly, 235Glu, 236Leu, 237Ser, 238Asn, 239Ser, 240Arg, 241Phe, 242Pro, Ala244Pro, 246Asp, 248Leu, 249Tyr, 250Thr, 251Asp, 252Pro, 254Glu, 256Ile, 257Val, 258Val, 262Asn, 263Gly, 264Arg, 265Cys, 266Thr, 267Leu, 268Asp, 269Gly, 270Thr, 271Leu, 272Gln, 273Gly, 276Gln, 277Leu, 278Val, 281Gln, 285Phe, 286Arg, 287Gly, 289Leu, 292Gln, 295Arg, 298Asp, 301Asp, Pro303Ala, 305Arg, 309His, 310Pro, 311Leu, 312His, 313Val, 318Leu, 320Gly, 323Tyr, 324Asp, 325Pro, 326Thr, Asp328Glu, Ile329Val, 330Pro, 331Ala, 333Leu, 334Gly, 337Asp, 338Phe, 339Lys, 340Gly, 341Thr, 342Val, 344Gly, 347Ser, 348Gln, 349Arg, 353Gly, 359His, 361Ala, 362Thr, 363Arg, 364Ala, 366Glu, 369Ile, 370Asp, 371Thr, 376Tyr, 379Lys, 380Leu, 381Gly, Ile383Val, 384Leu, 389Ser, 391Asp, 392Phe, 396Gln, 397Pro, 400Phe, 401Thr, 402Pro, Val403Ile, 404Gly, 406Gly, 412Gln, 415Leu, 416Pro, Asp417Glu, 418Tyr, 420Gly, 422Leu, 423Thr, 424Leu, 425Asn, 428Leu, 430Pro, 431Ala, 432Val, Ser433Ala, Ser435Ala, 436Phe, 437Pro, 440Arg, 441Ile, 442Leu, 444Phe, 445Arg, 446Ser, 448Val, 449Pro, 452Gly, 453Gly, 454Tyr, 455Gly, 456Ser, 457Gly, 460Asp, 466Glu, 470His, 472Tyr, 473Gln, 474Glu, 475Ala, 476Ala, 477Pro, 479Gln, 480Ser, 481Ala, 482Val, 483Ala, 484Leu, Val485Ile, 486Arg, 488Val, 489Asn, 490Pro, 491Asp, 494Arg, 499Ala, 500Lys, 501Leu, 502His, 504Glu, 505Gly, 510Ala, 513Gly, 514Asn, 515Asn, 516Pro, 518Val, 519Val, 521Pro, 523Gly, 524Tyr, 525Phe, 526Arg, 527Phe, 529Ala, 531Val, 532Asn, 533Gln, 534Phe, 535Tyr, 537Leu, 543Gly, 546Arg, 547Arg, 548Arg, 550Gln (n=298)

	MK956199	8Ala, 11Ser, 12Asn, 32Val, 37Ile, 38Ala, 41Val, 43Gly, 45Gln, 46Asn, 57Val, 62Gly, 64Phe, 65Thr, 66Val, 69Arg, 70Asn, 72Pro, 74Glu, 76Leu, 77Leu, 78Asn, 80Glu, 81Leu, 84Glu, 85Leu, 86Asn, 87Pro, 88Tyr, 89Leu, 91His, 92Leu, Pro94Arg, 96Tyr, 97Asn, 98Gly, 102Gly, 112Asn, 117Gly, 124Val, 128Phe, 129Pro, 130Val, Asp131Glu, 132Asn, 133Ile, 136Ala, 138Ile, 139Thr, 144Val, 148Val, 149arg, 150Gln, 152Glu, 154Val, 156Leu, 158Leu, 159Pro, 160Asp, 161Ile, 163Asn, 164Arg, 166Phe, 168Tyr, 169Asn, 171Glu, 172Asn, 173Thr, 175Arg, 177Arg, 178Leu, 179Val, 180Ala, 182Leu, 183Tyr, 184Thr, 185Pro, 186Leu, 187Arg, 188Ala, 189Asn, 191Gly, 193Asp, 195Phe, 196Thr, 197Val, 199Cys, 200Arg, 201Val, 202Leu, 203Thr, 204Arg, 205Pro, 207Pro, 208Asp, 210Glu, 211Phe, 212Thr, 213Phe, 214Leu, 215Val, 218Thr, 219Val, 220Glu, 223Thr, 224Lys, 225Pro, 226Phe, 228Leu, 229Pro, 230Ile, 231Leu, 232Thr, 234Gly, 235Glu, 236Leu, 237Ser, 238Asn, 239Ser, 240Arg, 241Phe, 242Pro, Ala244Pro, 246Asp, 248Leu, 249Tyr, 250Thr, 251Asp, 252Pro, 254Glu, 256Ile, 257Val, 258Val, 262Asn, 263Gly, 264Arg, 265Cys, 266Thr, 267Leu, 268Asp, 269Gly, 270Thr, 271Leu, 272Gln, 273Gly, 276Gln, 277Leu, 278Val, 281Gln, 285Phe, 286Arg, 287Gly, 289Leu, 292Gln, 295Arg, 298Asp, 301Asp, Pro303Ala, 305Arg, 309His, 310Pro, 311Leu, 312His, 313Val, 318Leu, 320Gly, 323Tyr, 324Asp, 325Pro, 326Thr, Asp328Glu, Ile329Val, 330Pro, 331Ala, 333Leu, 334Gly, 337Asp, 338Phe, 339Lys, 340Gly, 341Thr, 342Val, 344Gly, 347Ser, 348Gln, 349Arg, 353Gly, 359His, 361Ala, 362Thr, 363Arg, 364Ala, 366Glu, 369Ile, Asp370Asn, 371Thr, 376Tyr, 379Lys, 380Leu, 381Gly, 383Ile, 384Leu, 389Ser, 391Asp, 392Phe, 396Gln, 397Pro, 400Phe, 401Thr, 402Pro, Val403Ile, 404Gly, 406Gly, 412Gln, 415Leu, 416Pro, 417Asp, 418Tyr, 420Gly, 422Leu, 423Thr, 424Leu, 425Asn, 428Leu, 430Pro, 431Ala, 432Val, Ser433Ala, Ser435Ala, 436Phe, 437Pro, 440Arg, 441Ile, 442Leu, 444Phe, 445Arg, 446Ser, 448Val, 449Pro, 452Gly, 453Gly, 454Tyr, 455Gly, 456Ser, 457Gly, 460Asp, 466Glu, 470His, 472Tyr, 473Gln, 474Glu, 475Ala, 476Ala, 477Pro, 479Gln, 480Ser, 481Ala, 482Val, 483Ala, 484Leu, Val485Ile, 486Arg, 488Val, 489Asn, 490Pro, 491Asp, 494Arg, 499Ala, 500Lys, 501Leu, 502His, 504Glu, 505Gly, 510Ala, 513Gly, 514Asn, 515Asn, 516Pro, 518Val, 519Val, 521Pro, 523Gly, 524Tyr, 525Phe, 526Arg, 527Phe, 529Ala, 531Val, 532Asn, 533Gln, 534Phe, 535Tyr, 537Leu, 543Gly, 546Arg, 547Arg, 548Arg, 550Gln (n=298)
