# Supplementary material for: Molecular Evolutionary Analyses of the RNA-Dependent RNA Polymerase (RdRp) Region and VP1 Gene in Human Norovirus Genotypes GII.P6-GII.6 and GII.P7-GII.6
Source: Viruses. 2023 Jul 1;15(7):1497. doi: 10.3390/v15071497 (PMC10383674; doi:10.3390/v15071497)
Supplement: Supplementary file 1 [file viruses-15-01497-s001.zip › Table S2-4. VP1&RdRp_selection.zip!/Table S4. Datailed amino acid sequences of the VP1 gene for representive and prototype strains of each cluster used in this study..pdf]

Table S4. Detailed of the amino acid sequence of the *VP1* gene for the representative and prototype strains of each cluster in this study. Blue areas show conformational epitopes and green areas show Protruding 2 domain (aa279 to 406). The yellow boxes show positive selection sites.

	1	2	3	4	5	6	7	8	9	10	11	12	13	14	15	16	17	18	19	20	21	22	23	24	25	26	27	28	29	30
AB039777(P7)	M	K	M	A	S	N	D	A	A	P	S	N	D	G	A	A	N	L	V	P	E	A	N	N	E	V	M	A	L	E
LC122916(P7)	M	K	M	A	S	N	D	A	A	P	S	N	D	G	A	A	N	L	V	P	E	A	N	N	E	V	M	A	L	E
MK956199(P7)	M	K	M	A	S	N	D	A	A	P	S	N	D	G	A	A	N	L	V	P	E	A	N	N	E	V	M	A	L	E
MH791993(P7)	M	K	M	A	S	N	D	A	A	P	S	N	D	G	A	A	N	L	V	P	E	A	T	N	E	V	M	A	L	E
JX989075(P6)	M	K	M	A	S	N	D	A	A	P	S	N	D	G	A	A	N	L	V	P	E	A	N	N	E	V	M	A	L	E

	31	32	33	34	35	36	37	38	39	40	41	42	43	44	45	46	47	48	49	50	51	52	53	54	55	56	57	58	59	60
AB039777(P7)	P	V	V	G	A	S	I	A	A	P	V	V	G	Q	Q	N	I	I	D	P	W	I	R	E	N	F	V	Q	A	P
LC122916(P7)	P	V	V	G	A	S	I	A	A	P	V	V	G	Q	Q	N	I	I	D	P	W	I	R	E	N	F	V	Q	A	P
MK956199(P7)	P	V	V	G	A	S	I	A	A	P	V	V	G	Q	Q	N	I	I	D	P	W	I	R	E	N	F	V	Q	A	P
MH791993(P7)	P	V	V	G	A	S	I	A	A	P	V	V	G	Q	Q	N	I	I	D	P	W	I	R	E	N	F	V	Q	A	P
JX989075(P6)	P	V	V	G	A	S	I	A	A	P	V	V	G	Q	Q	N	I	I	D	P	W	I	R	E	N	F	V	Q	A	P

	61	62	63	64	65	66	67	68	69	70	71	72	73	74	75	76	77	78	79	80	81	82	83	84	85	86	87	88	89	90
AB039777(P7)	Q	G	E	F	T	V	S	P	R	N	S	P	G	E	M	L	L	N	L	E	L	G	P	E	L	N	P	Y	L	S
LC122916(P7)	Q	G	E	F	T	V	S	P	R	N	S	P	G	E	M	L	L	N	L	E	L	G	P	E	L	N	P	Y	L	S
MK956199(P7)	Q	G	E	F	T	V	S	P	R	N	S	P	G	E	M	L	L	N	L	E	L	G	P	E	L	N	P	Y	L	S
MH791993(P7)	Q	G	E	F	T	V	S	P	R	N	S	P	G	E	M	L	L	N	L	E	L	G	P	E	L	N	P	Y	L	G
JX989075(P6)	Q	G	E	F	T	V	S	P	R	N	S	P	G	E	M	L	L	N	L	E	L	G	P	E	L	N	P	Y	L	S

	91	92	93	94	95	96	97	98	99	100	101	102	103	104	105	106	107	108	109	110	111	112	113	114	115	116	117	118	119	120
AB039777(P7)	H	L	S	P	M	Y	N	G	Y	A	G	G	M	Q	V	Q	V	V	L	A	G	N	A	F	T	A	G	K	I	I
LC122916(P7)	H	L	S	R	M	Y	N	G	Y	A	G	G	M	Q	V	Q	V	V	L	A	G	N	A	F	T	A	G	K	I	I

MK956199(P7)	H	L	S	R	M	Y	N	G	Y	A	G	G	M	Q	V	Q	V	V	L	A	G	N	A	F	T	A	G	K	I	I
MH791993(P7)	H	L	S	R	M	Y	N	G	Y	A	G	G	M	Q	V	Q	V	V	L	A	G	N	A	F	T	A	G	K	I	I
JX989075(P6)	H	L	S	R	M	Y	N	G	Y	A	G	G	M	Q	V	Q	V	V	L	A	G	N	A	F	T	A	G	K	I	I

	121	122	123	124	125	126	127	128	129	130	131	132	133	134	135	136	137	138	139	140	141	142	143	144	145	146	147	148	149	150
AB039777(P7)	F	A	A	V	P	P	H	F	P	V	D	N	I	S	A	A	Q	I	T	M	C	P	H	V	I	V	D	V	R	Q
LC122916(P7)	F	A	A	V	P	P	H	F	P	V	D	N	I	S	A	A	Q	I	T	M	C	P	H	V	I	V	D	V	R	Q
MK956199(P7)	F	A	A	V	P	P	H	F	P	V	E	N	I	S	A	A	Q	I	T	M	C	P	H	V	I	V	D	V	R	Q
MH791993(P7)	F	A	A	V	P	P	H	F	P	V	E	N	I	S	A	A	Q	I	T	M	C	P	H	V	I	V	D	V	R	Q
JX989075(P6)	F	A	A	V	P	P	H	F	P	V	E	N	I	S	A	A	Q	I	T	M	C	P	H	V	I	V	D	V	R	Q

	151	152	153	154	155	156	157	158	159	160	161	162	163	164	165	166	167	168	169	170	171	172	173	174	175	176	177	178	179	180
AB039777(P7)	L	E	P	V	P	L	P	L	P	D	I	R	N	R	F	F	H	Y	N	Q	E	N	T	P	R	M	R	L	V	A
LC122916(P7)	L	E	P	V	L	L	P	L	P	D	I	R	N	R	F	F	H	Y	N	Q	E	D	T	P	R	M	R	L	V	A
MK956199(P7)	L	E	P	V	L	L	P	L	P	D	I	R	N	R	F	F	H	Y	N	Q	E	N	T	P	R	M	R	L	V	A
MH791993(P7)	L	E	P	V	L	L	P	L	P	D	I	R	N	R	F	F	H	Y	N	Q	E	N	T	P	R	M	R	L	V	A
JX989075(P6)	L	E	P	V	L	L	P	L	P	D	I	R	N	R	F	F	H	Y	N	Q	E	D	T	P	R	M	R	L	V	A

	181	182	183	184	185	186	187	188	189	190	191	192	193	194	195	196	197	198	199	200	201	202	203	204	205	206	207	208	209	210
AB039777(P7)	M	L	Y	T	P	L	R	A	N	S	G	E	D	V	F	T	V	S	C	R	V	L	T	R	P	A	P	D	F	E
LC122916(P7)	M	L	Y	T	P	L	R	A	N	S	G	E	D	V	F	T	V	S	C	R	V	L	T	R	P	A	P	D	F	E
MK956199(P7)	M	L	Y	T	P	L	R	A	N	S	G	E	D	V	F	T	V	S	C	R	V	L	T	R	P	A	P	D	F	E
MH791993(P7)	M	L	Y	T	P	L	R	A	N	S	G	E	D	V	F	T	V	S	C	R	V	L	T	R	P	A	P	D	F	E
JX989075(P6)	M	L	Y	T	P	L	R	A	N	S	G	E	D	V	F	T	V	S	C	R	V	L	T	R	P	A	P	D	F	E

	211	212	213	214	215	216	217	218	219	220	221	222	223	224	225	226	227	228	229	230	231	232	233	234	235	236	237	238	239	240
AB039777(P7)	F	T	F	L	V	P	P	T	V	E	S	K	T	K	P	F	T	L	P	I	L	T	L	G	E	L	S	N	S	R
LC122916(P7)	F	T	F	L	V	P	P	T	V	E	S	K	T	K	P	F	T	L	P	I	L	T	L	G	E	L	S	N	S	R

MK956199(P7)	F	T	F	L	V	P	P	T	V	E	S	K	T	K	P	F	T	L	P	I	L	T	L	G	E	L	S	N	S	R
MH791993(P7)	F	T	F	L	V	P	P	T	V	E	S	K	T	K	P	F	T	L	P	I	L	T	L	G	E	L	S	N	S	R
JX989075(P6)	F	T	F	L	V	P	P	T	V	E	S	K	T	K	P	F	T	L	P	I	L	T	L	G	E	L	S	N	S	R

	241	242	243	244	245	246	247	248	249	250	251	252	253	254	255	256	257	258	259	260	261	262	263	264	265	266	267	268	269	270
AB039777(P7)	F	P	A	A	I	D	M	L	Y	T	D	P	N	E	S	I	V	V	Q	P	Q	N	G	R	C	T	L	D	G	T
LC122916(P7)	F	P	A	A	I	D	M	L	Y	T	D	P	N	E	S	I	V	V	Q	P	Q	N	G	R	C	T	L	D	G	T
MK956199(P7)	F	P	A	P	I	D	M	L	Y	T	D	P	N	E	G	I	V	V	Q	P	Q	N	G	R	C	T	L	D	G	T
MH791993(P7)	F	P	A	P	I	D	M	L	Y	T	D	P	N	E	A	I	V	V	Q	P	Q	N	G	R	C	T	L	D	G	T
JX989075(P6)	F	P	A	P	I	D	M	L	Y	T	D	P	N	E	T	I	V	V	Q	P	Q	N	G	R	C	T	L	D	G	T

	271	272	273	274	275	276	277	278	279	280	281	282	283	284	285	286	287	288	289	290	291	292	293	294	295	296	297	298	299	300
AB039777(P7)	L	Q	G	T	T	Q	L	V	P	T	Q	I	C	A	F	R	G	T	L	I	S	Q	T	A	R	A	A	D	S	T
LC122916(P7)	L	Q	G	T	T	Q	L	V	P	T	Q	I	C	A	F	R	G	T	L	I	S	Q	N	A	R	A	A	D	L	T
MK956199(P7)	L	Q	G	T	T	Q	L	V	P	T	Q	I	C	A	F	R	G	T	L	I	G	Q	T	S	R	S	S	D	S	T
MH791993(P7)	L	Q	G	T	T	Q	L	V	P	T	Q	I	C	S	F	R	G	T	L	I	S	Q	T	S	R	S	A	D	S	T
JX989075(P6)	L	Q	G	T	T	Q	L	V	P	T	Q	I	C	S	F	R	G	T	L	I	S	Q	T	S	R	S	A	D	S	T

	301	302	303	304	305	306	307	308	309	310	311	312	313	314	315	316	317	318	319	320	321	322	323	324	325	326	327	328	329	330
AB039777(P7)	D	S	P	Q	R	A	R	D	H	P	L	H	V	Q	V	K	N	L	G	G	T	Q	Y	D	P	T	D	D	I	P
LC122916(P7)	D	S	P	Q	R	A	R	N	H	P	L	H	V	Q	V	K	N	L	D	G	T	Q	Y	D	P	T	D	D	I	P
MK956199(P7)	D	S	A	P	R	R	R	D	H	P	L	H	V	Q	L	K	N	L	D	G	T	Q	Y	D	P	T	D	E	V	P
MH791993(P7)	D	S	A	P	R	V	R	N	H	P	L	H	V	Q	L	K	N	L	D	G	T	P	Y	D	P	T	D	E	V	P
JX989075(P6)	D	S	A	P	R	V	R	N	H	P	L	H	V	Q	L	K	N	L	D	G	T	P	Y	D	P	T	D	E	V	P

	331	332	333	334	335	336	337	338	339	340	341	342	343	344	345	346	347	348	349	350	351	352	353	354	355	356	357	358	359	360
AB039777(P7)	A	V	L	G	A	I	D	F	K	G	T	V	F	G	V	A	S	Q	R	D	V	S	G	P	Q	E	Q	G	H	Y
LC122916(P7)	A	V	L	G	A	I	D	F	K	G	T	V	F	G	V	A	S	Q	R	D	V	S	G	T	Q	E	Q	G	H	Y

MK956199(P7)	A	V	L	G	A	I	D	F	K	G	T	V	F	G	V	A	S	Q	R	D	V	S	G	Q	Q	V	G	-	-	-
MH791993(P7)	A	V	L	G	A	I	D	F	K	G	T	V	F	G	I	A	S	Q	R	N	T	T	G	S	S	I	G	-	-	-
JX989075(P6)	A	V	L	G	A	I	D	F	K	G	T	V	F	G	V	A	S	Q	R	N	T	T	G	S	S	V	G	-	-	-
	361	362	363	364	365	366	367	368	369	370	371	372	373	374	375	376	377	378	379	380	381	382	383	384	385	386	387	388	389	390
AB039777(P7)	A	T	R	A	H	E	A	H	I	D	T	T	D	P	K	Y	A	P	K	L	G	T	I	L	I	K	S	E	S	N
LC122916(P7)	A	T	R	A	H	E	A	H	I	D	T	T	D	P	K	Y	A	P	K	L	G	T	I	L	I	K	S	E	S	T
MK956199(P7)	A	T	R	A	H	E	V	H	I	N	T	T	D	P	R	Y	T	P	K	L	G	S	I	L	M	H	S	E	S	D
MH791993(P7)	A	T	R	A	H	E	V	H	I	D	T	T	N	P	R	Y	T	P	K	L	G	S	V	L	M	Y	S	E	S	N
JX989075(P6)	A	T	R	A	H	E	V	H	I	D	T	T	N	P	R	Y	T	P	K	L	G	S	V	L	M	H	S	E	S	S
	391	392	393	394	395	396	397	398	399	400	401	402	403	404	405	406	407	408	409	410	411	412	413	414	415	416	417	418	419	420
AB039777(P7)	D	F	T	T	N	Q	P	I	R	F	T	P	V	G	M	G	D	N	N	W	R	Q	W	E	L	P	D	Y	S	G
LC122916(P7)	D	F	T	T	N	Q	P	I	R	F	T	P	V	G	M	G	D	N	N	W	R	Q	W	E	L	P	D	Y	S	G
MK956199(P7)	D	F	V	T	G	Q	P	V	R	F	T	P	I	G	M	G	D	N	D	W	H	Q	W	E	L	P	D	Y	S	G
MH791993(P7)	D	F	D	D	G	Q	P	T	R	F	T	P	I	G	M	G	A	D	D	W	R	Q	W	E	L	P	E	Y	S	G
JX989075(P6)	D	F	D	D	G	Q	P	T	R	F	T	P	I	G	M	G	A	D	D	W	H	Q	W	E	L	P	E	Y	S	G
	421	422	423	424	425	426	427	428	429	430	431	432	433	434	435	436	437	438	439	440	441	442	443	444	445	446	447	448	449	450
AB039777(P7)	R	L	T	L	N	M	N	L	A	P	A	V	S	P	S	F	P	G	E	R	I	L	F	F	R	S	I	V	P	S
LC122916(P7)	S	L	T	L	N	M	N	L	A	P	A	V	S	P	S	F	P	G	E	R	I	L	F	F	R	S	I	V	P	S
MK956199(P7)	R	L	T	L	N	M	N	L	A	P	A	V	A	P	A	F	P	G	E	R	I	L	F	F	R	S	I	V	P	S
MH791993(P7)	H	L	T	L	N	M	N	L	A	P	A	V	A	P	A	F	P	G	E	R	I	L	F	F	R	S	V	V	P	S
JX989075(P6)	H	L	T	L	N	M	N	L	A	P	A	V	A	P	A	F	P	G	E	R	I	L	F	F	R	S	V	V	P	S
	451	452	453	454	455	456	457	458	459	460	461	462	463	464	465	466	467	468	469	470	471	472	473	474	475	476	477	478	479	480
AB039777(P7)	A	G	G	Y	G	S	G	Y	I	D	C	L	I	P	Q	E	W	V	Q	H	F	Y	Q	E	A	A	P	S	Q	S
LC122916(P7)	A	G	G	Y	G	S	G	Y	I	D	C	L	I	P	Q	E	W	V	Q	H	F	Y	Q	E	A	A	P	S	Q	S

MK956199(P7)	A	G	G	Y	G	S	G	Q	I	D	C	L	I	P	Q	E	W	V	Q	H	F	Y	Q	E	A	A	P	S	Q	S
MH791993(P7)	A	G	G	Y	G	S	G	H	I	D	C	L	I	P	Q	E	W	V	Q	H	F	Y	Q	E	A	A	P	S	Q	S
JX989075(P6)	A	G	G	Y	G	S	G	Q	I	D	C	L	I	P	Q	E	W	V	Q	H	F	Y	Q	E	A	A	P	S	Q	S

	481	482	483	484	485	486	487	488	489	490	491	492	493	494	495	496	497	498	499	500	501	502	503	504	505	506	507	508	509	510
AB039777(P7)	A	V	A	L	V	R	Y	V	N	P	D	T	G	R	N	I	F	E	A	K	L	H	R	E	G	F	L	T	V	A
LC122916(P7)	A	V	A	L	V	R	Y	V	N	P	D	T	G	R	N	I	F	E	A	K	L	H	R	E	G	F	L	T	V	A
MK956199(P7)	A	V	A	L	I	R	Y	V	N	P	D	T	G	R	N	I	F	E	A	K	L	H	R	E	G	F	I	T	V	A
MH791993(P7)	A	V	A	L	I	R	Y	V	N	P	D	T	G	R	N	I	F	E	A	K	L	H	R	E	G	F	I	T	V	A
JX989075(P6)	A	V	A	L	I	R	Y	V	N	P	D	T	G	R	N	I	F	E	A	K	L	H	R	E	G	F	I	T	V	A

	511	512	513	514	515	516	517	518	519	520	521	522	523	524	525	526	527	528	529	530	531	532	533	534	535	536	537	538	539	540
AB039777(P7)	N	S	G	N	N	P	I	V	V	P	P	N	G	Y	F	R	F	E	A	W	V	N	Q	F	Y	T	L	A	P	M
LC122916(P7)	N	S	G	N	N	P	I	V	V	P	P	N	G	Y	F	R	F	E	A	W	V	N	Q	F	Y	T	L	A	P	M
MK956199(P7)	N	S	G	N	N	P	I	V	V	P	P	N	G	Y	F	R	F	E	A	W	V	N	Q	F	Y	T	L	T	P	M
MH791993(P7)	N	S	G	N	N	P	I	V	V	P	P	N	G	Y	F	R	F	E	A	W	V	N	Q	F	Y	T	L	T	P	M
JX989075(P6)	N	S	G	N	N	P	I	V	V	P	P	N	G	Y	F	R	F	E	A	W	V	N	Q	F	Y	T	L	T	P	M

	541	542	543	544	545	546	547	548	549	550
AB039777(P7)	G	S	G	Q	G	R	R	R	A	Q
LC122916(P7)	G	S	G	Q	G	R	R	R	A	Q
MK956199(P7)	G	T	G	Q	G	R	R	R	N	Q
MH791993(P7)	G	T	G	Q	G	R	R	R	V	Q
JX989075(P6)	G	T	G	Q	G	R	R	R	V	Q
